# DNA Carrier-Assisted Molecular Ping-Pong in an Asymmetric Nanopore

**DOI:** 10.1021/acs.nanolett.3c03605

**Published:** 2023-11-30

**Authors:** Fei Zheng, Mohammed Alawami, Jinbo Zhu, Casey M. Platnich, Jingjie Sha, Ulrich F. Keyser, Kaikai Chen

**Affiliations:** † Cavendish Laboratory, University of Cambridge, CB3 0HE Cambridge, United Kingdom; ‡ School of Nanoscience and Nanotechnology, University of Chinese Academy of Sciences, Beijing 101408, China; § Jiangsu Key Laboratory for Design and Manufacture of Micro-Nano Biomedical Instruments, School of Mechanical Engineering, Southeast University, Nanjing 211100, China; ∥ School of Biomedical Engineering, Faculty of Medicine, Dalian University of Technology, Dalian 116024, China

**Keywords:** molecular ping-pong, asymmetric nanopore, DNA carrier, flip dynamics

## Abstract

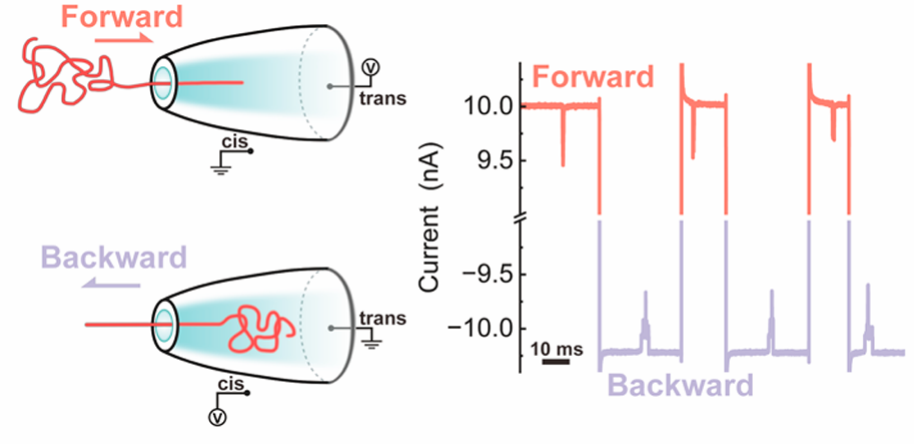

Nanopore analysis relies on ensemble averaging of translocation signals obtained from numerous molecules, requiring a relatively high sample concentration and a long turnaround time from the sample to results. The recapture and subsequent re-reading of the same molecule is a promising alternative that enriches the signal information from a single molecule. Here, we describe how an asymmetric nanopore improves molecular ping-pong by promoting the recapture of the molecule in the trans reservoir. We also demonstrate that the molecular recapture could be improved by linking the target molecule to a long DNA carrier to reduce the diffusion, thereby achieving over 100 recapture events. Using this ping-pong methodology, we demonstrate its use in accurately resolving nanostructure motifs along a DNA scaffold through repeated detection. Our method offers novel insights into the control of DNA polymer dynamics within nanopore confinement and opens avenues for the development of a high-fidelity DNA detection platform.

Nanopore sensing has undergone significant advancements in the past two decades, as this single-molecule platform exhibits promising technological applications, particularly in diagnostics.^1−5^ In a typical nanopore sensing experiment, a charged target is driven by an applied voltage across the nanopore, translocating from the cis to the trans side, with the recorded current reflecting the analyte’s features, such as the size and charge. Although simple structures yield easily distinguishable current–time trajectories, macromolecules such as DNA polymers frequently adopt intricate conformations upon entering the nanopore,^6−9^ adding complexity to signal interpretation. Hence, one must measure hundreds of individual translocation events of the same target molecules before statistical analysis, increasing the required sample amount and the time needed for measurement and analysis.

By repeatedly capturing or trapping the same molecule within a nanopore, a technique colloquially referred to as “molecular ping-pong”,^10^ makes it possible to analyze a single target molecule multiple times, thereby reducing the amount of sample required for an accurate readout.^11,12^ The most straightforward method to recapture DNA molecules involves promptly reversing the voltage polarity, right after translocation. As long as the molecule has not diffused far from the vicinity of the nanopore, within what we refer to as the capture area, it will be recaptured and translocated in the opposite direction. Hence, one cycle of recapture comprises both a forward translocation event and a subsequent backward translocation event.

Previous studies have shown that more recaptures can be achieved by minimizing the time interval between the end of the blockade event and the voltage reversal.^11^ Alternatively, a molecule may be trapped in a “tug-of-war” by two parallel nanopores^13−17^ or using optical tweezers.^18−20^ In each of these scenarios, the voltage difference between the two pores can be adjusted to shuttle the DNA molecule back and forth, facilitating multiple readings of the resultant signal. To date, most re-reading systems are based on symmetric nanopore structures,^11,12^ where symmetric opening areas are at both sides of the membrane nanopore. This symmetric feature also exists in the two-nanopore system of the same size within the tug-of-war configuration.^13−16^ This symmetry leads to the same recapture behavior for the forward and backward translocation. Our previous study using an asymmetric nanopore demonstrated that DNA polymers showed distinct translocation dynamics in opposite translocation directions,^19^ especially in the velocity profiles as a function of time. However, recapture within these nanopores has never been investigated, potentially indicating distinct behavior compared to symmetric nanopores.

In addition, designing an additional polymer chain, to which the target attached, has proven to be an effective way to improve the analysis using nanopores. For instance, DNA carriers were used to detect the size and shape of various DNA nanostructures,^21^ including protrusions,^22^ hairpins,^23^ dumbbells,^24−27^ rings,^28^ cubes,^3,28^ tetrahedrons,^29,30^ helix bundles,^31,32^ and multiway junctions,^33^ facilitating the advancement of DNA data storage,^34,35^ artificial nanoscale rotary motors,^36^ and respiratory infection diagnosis.^5^ The design of the DNA carrier may offer a smart strategy for improving molecular ping-pong by increasing the recapture probability.

Here, we demonstrate the use of an asymmetric nanopore for molecular ping-pong. We showed that the confinement in a conical nanopore facilitates DNA recapture, which restricts the diffusion of DNA. We obtained a 100% recapture of the molecule when the DNA was pulled back after entering the nanopore confinement. By attaching the target to a long 48.5 kb DNA molecule, we successfully enhanced the likelihood of recapturing short DNA targets. Our system marks a significant step toward “single-molecule re-reading” and demonstrates how the conical geometry and the additional DNA carrier can assist molecular ping-pong, with promising applications in high-accuracy analysis of short DNA fragments, as well as structured motifs along a DNA polymer.

Figure 1 illustrates the experimental setup of our molecular ping-pong method. Asymmetric, conical glass nanopores were used in all experiments, with diameters of 14 ± 3 nm (mean ± standard deviation (s.d.)) and cone semiangle of 0.05 ± 0.01 radians (mean ± s.d.) based on a previous characterization.^24^ First, the DNA molecule initiates a forward translocation (Figure 1a). Once the translocation signal is detected by the ping-pong setup, it triggers the voltage polarity reversal. Subsequently, the reversed voltage will force the molecule to translocate backward (Figure 1b). Here, we define that one cycle of ping-pong comprises one forward event when the DNA translocates from the cis side to the trans side (cis–trans; Figure 1a) and one backward event with translocation in the opposite direction (trans–cis) (Figure 1b). Figure 1c shows the current trace of a typical recapture process. One forward event corresponds to a downward spike signal in the current–time trace at 600 mV and, in contrast, the backward one appears as an upward spike signal at −600 mV.

**Figure 1:**
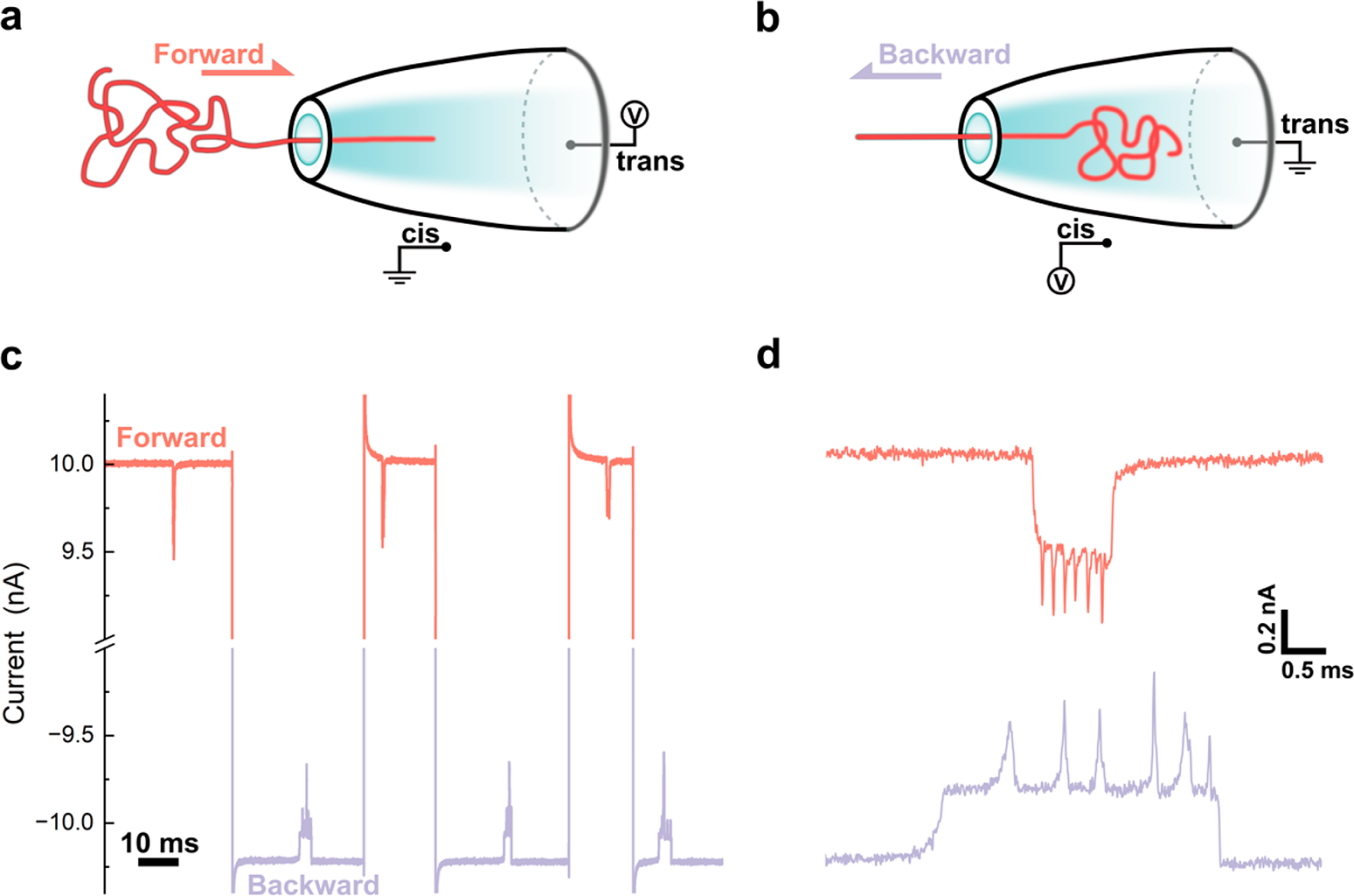
Schematic and signals of molecular ping-pong in an asymmetric conical nanopore. (a) Schematic of the forward translocation. A +600 mV voltage was applied at the trans side. (b) Schematic of the backward translocation. A −600 mV voltage was applied at the trans side. (c) Example segment of the ping-pong process with 3 recapture cycles of a 7.2 kb dsDNA molecule. (d) Enlarged view of forward and backward translocation signals. The translocation time of the backward signal was approximately 3 times as long as the forward signal. The experiment was conducted in 4 M LiCl solution with 10 mM Tris (pH 8.0). The DNA concentration is ∼18 pM (translocation frequency is ∼4 events/min).

We initially examined ping-pong using a double-stranded DNA (dsDNA) molecule that measured 7228 bp in length, and it featured six evenly distributed groups of dumbbell structures attached to the scaffold. This structure is commonly referred to as the “DNA marker”.^19^ This DNA marker was assembled by adding complementary oligonucleotides to the single-stranded scaffold from phage M13mp18. The detail of different types of DNA molecules used in this work can be found in Supporting Information Table S8. Examples of single forward and backward events are shown in Figure 1d. Six additional spikes are clearly observed in both the forward and backward events, establishing that the ping-pong method can be used to detect a single DNA structure repeatedly. In contrast to symmetric membrane nanopores, where forward and backward translocations exhibited similar signals, here the backward translocation time is much longer than the forward one. For our 14 nm nanopores, the backward translocation velocity was roughly one-third of the forward translocation velocity.^19^

By using the conical geometry of the nanopore, we created two distinct regions in which DNA molecules exhibit different dynamics. For this asymmetric nanopore, outside the conical nanopore confinement is the open “cis” reservoir, and the inside of the nanopore is referred to as the “trans” reservoir, as illustrated in Figure 2a. In the region distant from the nanopore on the cis side, DNA motion is primarily governed by diffusion.^37^ Once DNA diffuses within the access region of the nanopore, it is captured by the electric field and then driven through the nanopore (forward translocation). When the DNA molecule enters the nanopore, diffusion is restricted due to the confinement of space, formed by the cone with a 14 nm diameter at one end, expanding with a conical angle of 0.05 radians.^19^ As such, a voltage reversal will easily recapture a restricted molecule and force it back through the nanopore. Subsequently, after a backward translocation, when the molecule returns to the open (cis) space, it can diffuse in many directions with a high probability of escaping from the nanopore. Therefore, the ping-pong strategy in asymmetric nanopores achieved more recaptures than in symmetric ones, where both sides are open space.

**Figure 2:**
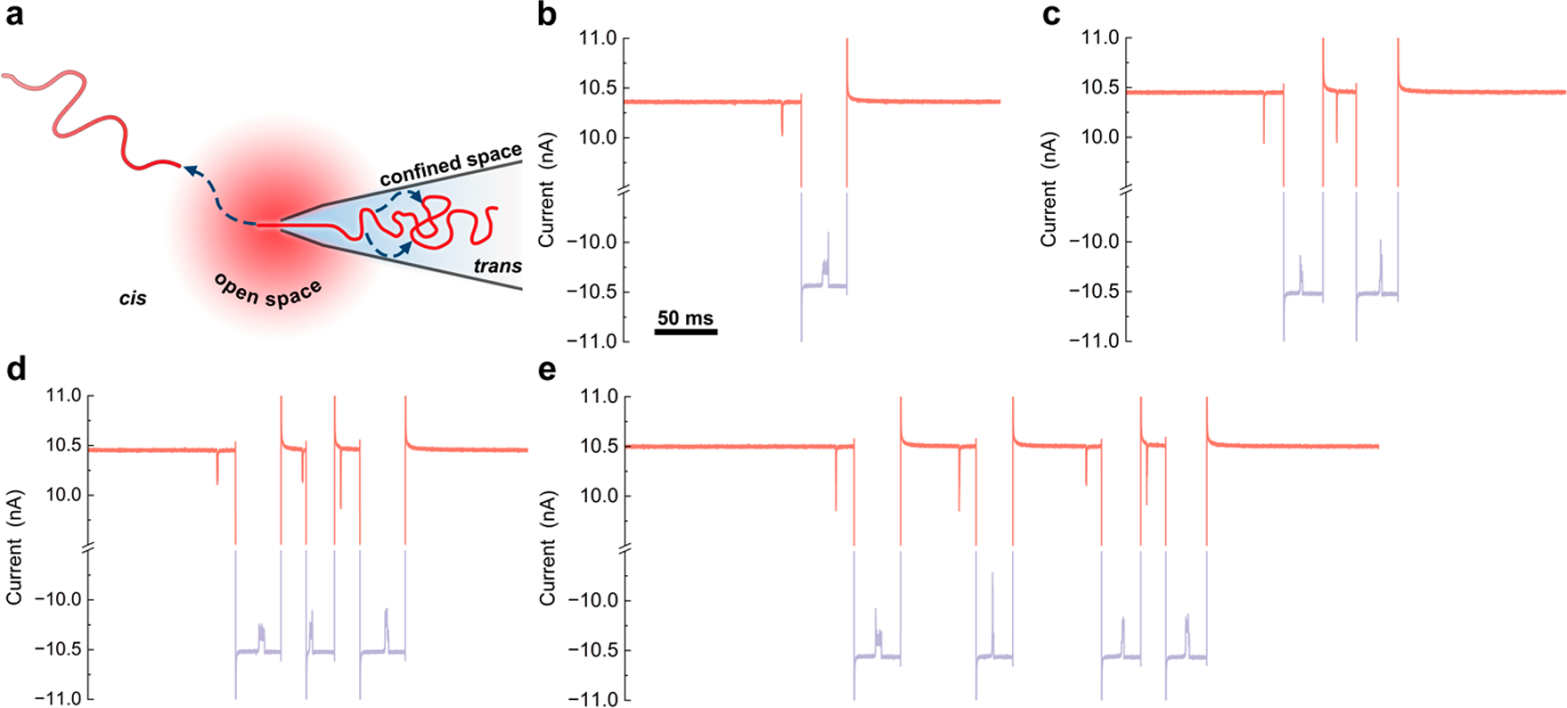
DNA molecule 100% recaptured at the trans side. (a) Schematic of the mechanism of recapture at the trans side (confined space) and escape at the cis side (open space). (b–e) Examples of 1–4 cycles of recapture of 8 kb DNA molecules (do not modify with dumbbell structures). The molecule consistently underwent recapture in the backward (trans–cis) direction while escaping in the forward (cis–trans) direction.

As expected, we found that in all ping-pong events, 100% of DNA molecules (Figure S1) were recaptured after the voltage was reversed to initiate backward translocation, higher than the 60% reported in symmetric nanopores with similar DNA length and delay time.^11^ We confirmed this result from over 10000 ping-pong processes recorded in 51 different pores with diameters of 14 ± 3 nm. We observed a 100% recapture rate on the trans side for DNA of varying lengths, ranging from 7.2 to 55.7 kb (Figure S2). After several recaptures, DNA molecules were finally lost at the cis side, ending the ping-pong. We illustrate this asymmetrical recapture feature in Figure 2b–e with four ping-pong examples, each showcasing the molecule being recaptured with 1, 2, 3, and 4 cycles, respectively.

We initially refined the ping-pong method by optimizing the onset time of voltage polarity switching to recapture the DNA molecule to increase the number of recaptures. Specifically, we programmed a custom LabVIEW code to accurately control the applied voltage in response to the recorded current signals. Once a current blockade is detected, the program reverses the voltage after a delay time, *T*_delay_ (Figure 3a). For the purpose of accurate signal analysis, we adjusted *T*_delay_ to ensure that the following event occurred only after completion of the current relaxation (Figure 3a). The relaxation of the ionic current to the baseline is inevitable due to the finite response time of the electrodes and amplifier after reversing the voltage quickly (see, for example, Figure 2b–e). By employing a 4 M LiCl solution, we decreased the relaxation time to less than 1 ms, resulting in a faster response compared to the 1 M KCl solution.^11^

**Figure 3:**
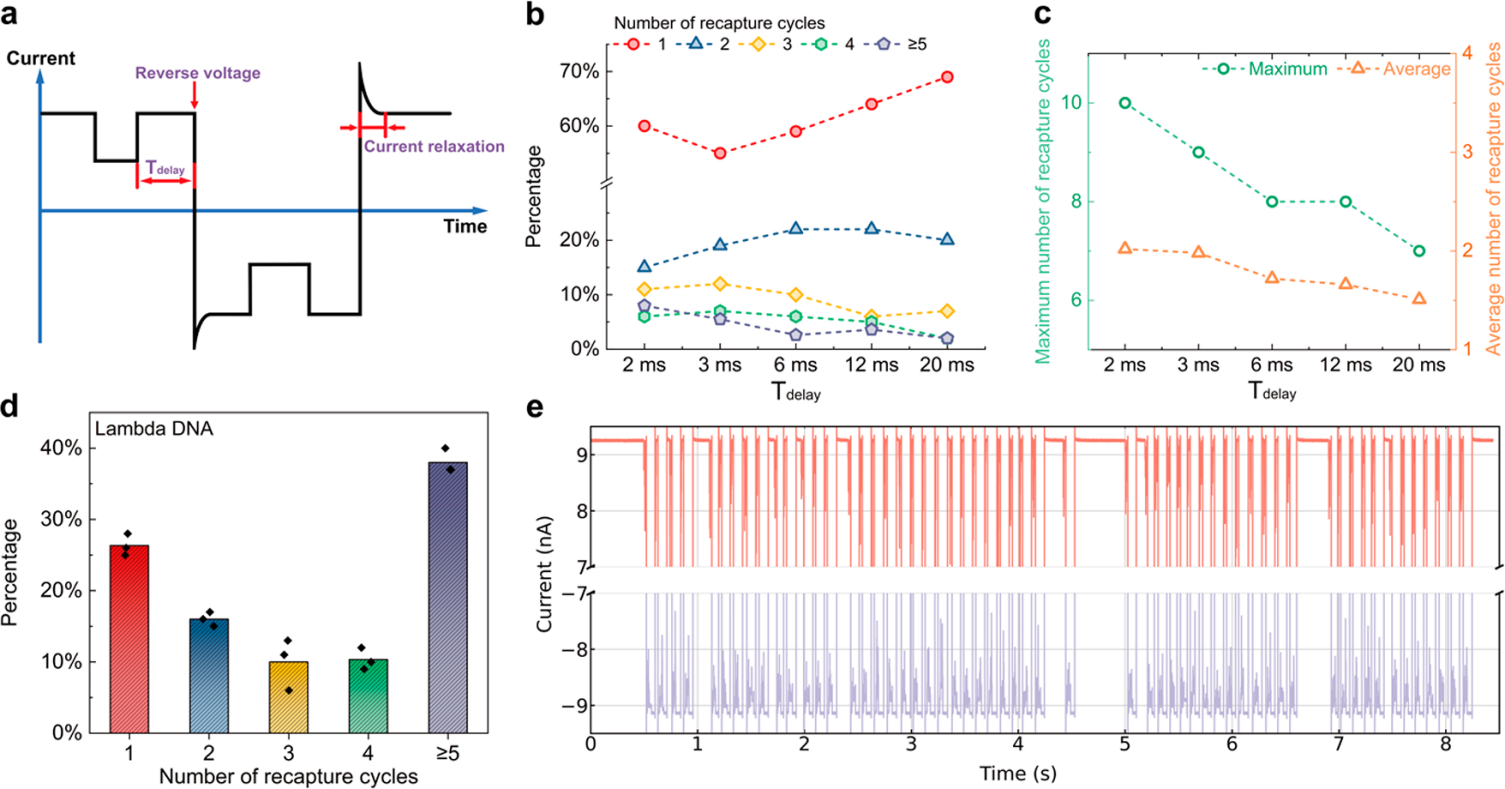
Influence factors of recaptures in ping-pong. (a) Schematic of the voltage control logic in the ping-pong method. (b) Percentages of different numbers of recapture cycles with varied delay time *T*_delay_ ranging from 2 to 20 ms for 8 kb DNA molecules derived from the same nanopore. (c) Maximum number of recapture cycles (green) and average number of recapture cycles (orange) as a function of *T*_delay_ for the 8 kb DNA molecule derived from the same nanopore. (d) Percentages of different numbers of recapture cycles for the lambda DNA (48.5 kb) in 3 similar-sized nanopores (*T*_delay_ set to 20 ms for forward translocations and 60 ms for backward translocations to ensure that each translocation during ping-pong was complete, confirmed by the power density spectrum (PSD) before and after translocations and the event charge deficit (ECD) of all events (Figures S6 and S7)). (e) Example of 57 cycle (114 captures) recaptures for a lambda DNA. We used a DNA concentration of ∼20 pM (∼4 events/min for the translocation frequency, Figure S11) to reduce the probability of capturing a second molecule.

We investigated how *T*_delay_ influenced the observed number of recaptures. Five *T*_delay_ values (20, 12, 6, 3, and 2 ms) were examined. For each *T*_delay_, we recorded for 1 h using the 8 kb DNA molecules and then calculated the percentage of different numbers of recaptures within each ping-pong process. We found that decreasing *T*_delay_ can increase the proportion of multiple-time recapture (Figure 3b, Table S4), indicating that a more rapid reversal of voltage polarity resulted in an increased probability of recapture, as expected and shown in a previous study.^11^ This observation is further supported by the maximum and average number of recapture cycles (Figure 3c), both of which exhibited an increase as *T*_delay_ was reduced. For the 8 kb DNA, the maximum number of recapture cycles was 10 at an optimized *T*_delay_ of 2 ms. The increment of recaptures resulting from a reduced *T*_delay_ is not as pronounced as expected, which might be caused by the limited range of delay times in our experiment. For the 8 kb DNA, the molecule was recaptured only for 2 cycles on average, limiting the use of this strategy in practical applications^33^ that usually require at least 10 reads of a molecule. One potential approach to enhance recapturing is to decrease the delay time more. However, this strategy may result in translocations occurring on the slope of the current–time trace, which can impact the analysis of molecular signals.

To improve the recapturing, we reduced the diffusion coefficient by adjusting the length of the DNA molecule. The diffusion coefficient directly impacts the escape probability for a DNA molecule at the open space (cis), with fast diffusion increasing the likelihood of escaping from the capture region. As the diffusion coefficient of a DNA molecule decreases with its length, here we used longer lambda DNA (48.5 kb) to reduce the diffusion coefficient. The same delay time, *T*_delay_ = 4 ms, was examined at first, but we observed that this long molecule was getting trapped inside the nanopore; i.e., the molecule did not completely exit the nanopore before it was recaptured again. In this situation, the full translocation signal was not successfully recorded. This trapping behavior is attributed to the fact that *T*_delay_ was relatively short compared to the translocation time that a lambda DNA molecule required to move outside the nanopore. Therefore, one must tune *T*_delay_ according to the translocation time of the target molecule. Considering this, we prolonged *T*_delay_ for lambda DNA and set different *T*_delay_ values for cis–trans (20 ms) and trans–cis (60 ms) translocations to avoid events during the current relaxation. After these optimizations, we obtained a higher proportion of multiple-time recapture for lambda DNA, for example, 33% for recapturing more than 5 cycles (Figure 3d, Table S7) compared with 8 kb DNA, 1.8% (Figure 3b, Table S4). The average number of recapture cycles for lambda DNA was 7, more than three times that of 8 kb DNA (average of 2). More importantly, the maximum number of cycles for lambda DNA reached 57 (114 captures, Figure 3e and Figure S8) with optimized delay time, approximately 6 times that for 8 kb DNA and roughly double the maximum recaptures achieved with lambda DNA monomer using symmetric nanopores.^12^ Apart from tuning the diffusion, other methods did not improve the recapture (Tables S5 and S6 and Figures S3–S5).

Although longer DNA molecules such as lambda can be recaptured and detected tens of times, multiple recaptures remained challenging for short DNA strands due to their faster diffusion. To solve this, we slowed the diffusion of short DNA molecules and recaptured them more than 100 times with the assistance of lambda DNA as a carrier. As a proof-of-concept, we linked the marker, a short 7.2 kb dsDNA, to the sticky end (12 nt overhang) of a lambda DNA by a link oligo, forming a construct (lambda + marker). Materials and methods are given in the Supporting Information.

As illustrated in Figure 4a, the 5′ of the link oligo is complementary to the 12 nt overhang at the 3′ of lambda DNA, while the 3′ is complementary to the 30 nt overhang at the 5′ end of the M13mp18 scaffold. On this scaffold, we also designed 6 groups of dumbbell structures as a feature to distinguish the translocation direction. Upon testing this linked construct, we observed six additional spikes in the translocation event (Figure 4b, green spikes). We tested this design in ping-pong and successfully detected the dumbbell structures in both forward and backward translocation events (Figure 4b). By means of this linking method, we can achieve similar numbers of recapture events for shorter DNA molecules to those for lambda DNA.

**Figure 4:**
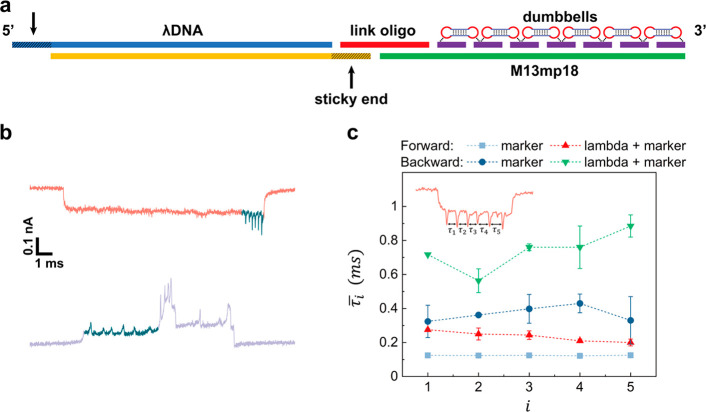
Analysis of target DNA assisted with a lambda carrier by molecular ping-pong. (a) Design of the linkage of a structured “cargo” (DNA marker) with a carrier (lambda DNA) using a link oligo with its 5′ end complementary to the 3′ end of the lambda DNA and the 3′ end complementary to the 5′ of the M13mp18 scaffold (not drawn to scale). The blue and yellow strands depict the double-stranded lambda DNA, featuring two shaded sticky ends. The red strand represents the single-stranded link oligo. The green strand represents the single-stranded M13mp18 with 190 purple oligos complementary to it. The dumbbells represent the 6 × 8 dumbbell structures (each dumbbell site has 8 dumbbell structures). (b) Examples of a forward (red) and backward (purple) translocation signal of a lambda + marker construct. (c) Translocation time interval between adjacent dumbbell structures, *τ*_i_, for the single DNA marker and the lambda + marker in the forward and backward translocation, respectively. Error bars show s.d. and were calculated by averaging over 5 events in a single nanopore.

In addition to slowing the diffusion, this linkage also reduced the translocation velocity of the short DNA molecule inside the nanopore. The velocity of the translocating molecule can be extracted using the evenly spaced 6 groups of dumbbells on the M13mp18 scaffold. We calculated the time interval between each 2 adjacent dumbbell spikes, shown as *τ*_i_ (the inset in Figure 4c). This time interval is inversely proportional to the temporal translocation velocity of the molecule.^25^ We then compared the velocities of a single DNA marker and a DNA marker bound to a lambda carrier for both the forward and backward cases (Figure 4c). *τ*_i_ values of a lambda + marker were all approximately 2 times of that of a single DNA marker, for both forward and backward translocations. This means the translocation velocity halved because of the extra hydrodynamic drag from the lambda carrier.^19^ Consequently, the nanopore measurement can be used to identify the nanostructures on the DNA molecule with greater accuracy. Additionally, *τ*_i_ of backward events were nearly 4 times that of forward events, suggesting that this accuracy can be further improved by employing ping-pong in an asymmetric nanopore. Examples of molecular ping-pong for the lambda + marker construct are shown in Figures S9 and S10.

Apart from the accurate recognition of DNA structures, this linkage also enables the tracking of dynamics for the same DNA molecule during the ping-pong process. The nanopore signals revealed that the DNA can flip during the recapture process, and we were able to study the dynamics of this process (Figure 5a,b). The asymmetric design of the linked molecule facilitated the monitoring of the flipping process, as only the 5′ of the DNA marker was linked to the 3′ of the lambda carrier; the two ends of the entire molecule (5′ end and 3′ end) were distinguishable in the translocation signals. As illustrated in Figure 5b, a typical flip in the ping-pong event proceeds as follows: In the first forward translocation, the DNA molecule enters with the 3′ end (marker, 6 dumbbell spikes) first, and upon reversing the voltage, the molecule comes back with the 5′ end (lambda) in the first backward translocation. Then, while reversing the voltage a second time, in the next forward translocation, the DNA molecule enters with the 5′ end (lambda) at the beginning and returns with the 3′ end (marker) at first. The corresponding current signals are plotted in Figure 5a. We found that flips always occurred at the cis side (9 flip events in total 13 ping-pong events recorded in 2 nanopore experiments), where the DNA molecule has more freedom to adopt an alternative configuration before recapture. We did not observe any flips in the trans side (0 flip event in 13 ping-pong events), where we attribute this behavior to the highly restricted space. The flip phenomenon demonstrates that molecular ping-pong can be a valuable tool for elucidating the structural dynamics of DNA polymers in and out of the confinement.

**Figure 5:**
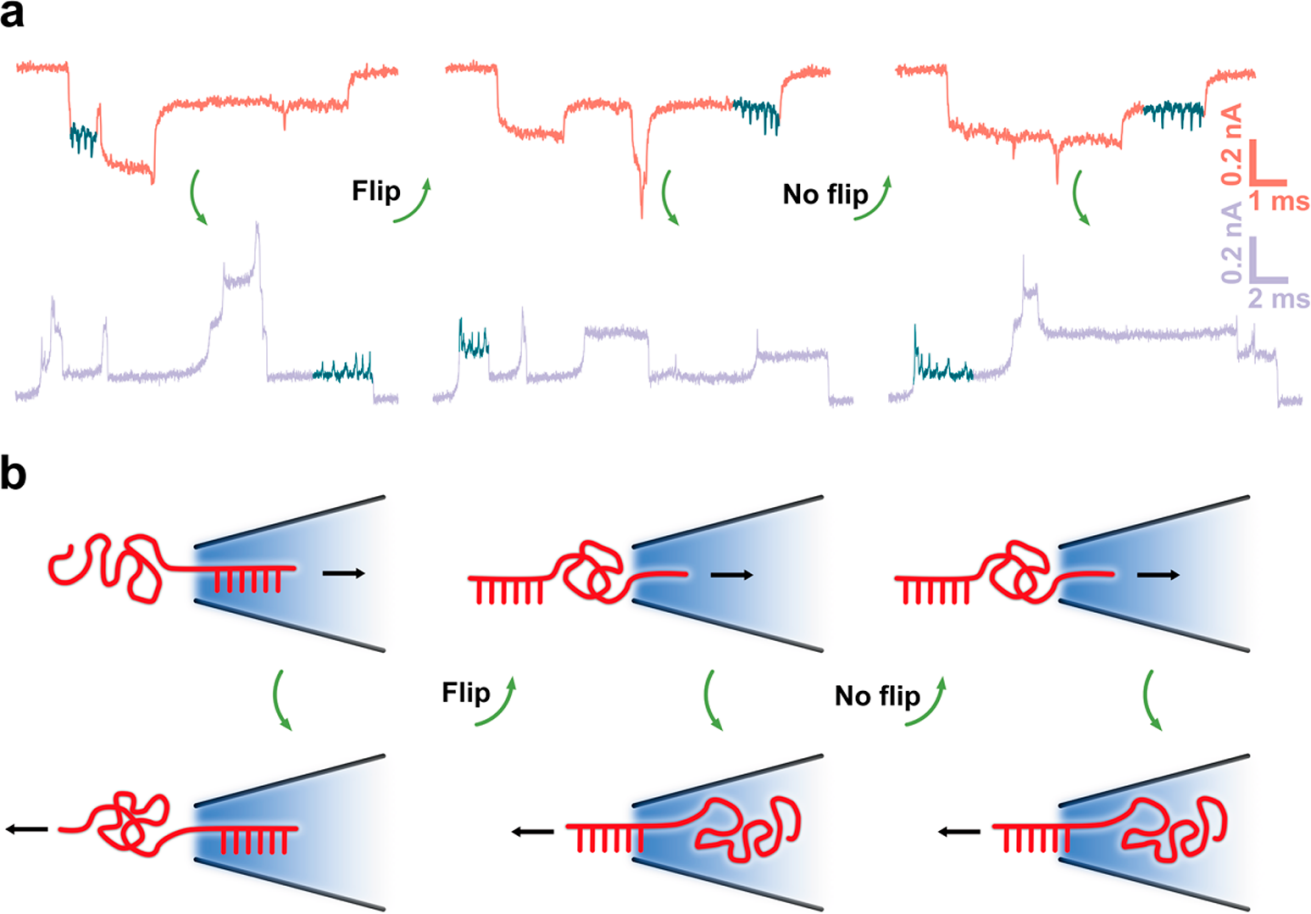
Flip dynamics in ping-pong. (a) Translocation signals during the flip. (b) Schematic of a flip and nonflip behavior. Conformations such as folds and knots are not drawn in the illustration.

When using the ping-pong method to re-read DNA structures, the signals of designed structures may be muddled due to natural DNA conformations like folds and knots, which also result in current drops and make the signals difficult to decipher. We expect that tail retraction of the entire polymer would facilitate linear translocation of the structured marker at the end. Furthermore, the delay time of ping-pong may be adjusted to approach the Zimm relaxation time,^25^ meaning the DNA molecule is given sufficient time to recover its equilibrium state, thereby untangling complex conformations.

To conclude, we investigated the use of asymmetric nanopores and a linkage strategy to facilitate the recapture and the re-reading of DNA molecules. Capitalizing on the nanopore’s asymmetry, the platform we designed traps the DNA molecule in the confined space of the trans reservoir, facilitating the repeated recapture of DNA molecules. Appending DNA nanostructures to the scaffold strands, we demonstrated multiple re-reads, paving the way for accurate detection of DNA nanostructures. As an added benefit, the translocation of the overall construct is slowed, enabling a more accurate readout of the molecular barcodes.

To further optimize the system and obtain more recapture events, the diffusion coefficient of the DNA molecule may be further decreased. For example, using two complementary sticky ends, the lambda DNA could self-connect into dimer, trimer, or even multimers after incubation. By appending short, target DNA to these multimers, we may capitalize on even lower diffusion coefficients to facilitate greater amounts of recapture. The anticipated efficiency might not be achieved, as the lambda DNA can self-connect into circular molecules, hindering the linkage with other entities.

The tuning strategy of diffusion here primarily focuses on modifying the length or size of DNA molecules through three different methods: binding to a long DNA carrier (lambda DNA), assembling dumbbell structures, and binding streptavidin to the scaffold. Among these methods, binding to a lambda carrier proves to be the most efficient way to improve the recapture efficiency. This is due to the significant increase in the entire molecule’s length with lambda DNA (48.5 kb) compared to the dumbbell structure (14 bp) and streptavidin (∼5 nm in diameter). Note that at the trans side diffusion is strictly confined for all three DNA types.

Importantly, ping-pong allowed us to observe as-of-yet unstudied conformational changes during translocations into and out of the confined space. For example, we have observed new flip dynamics, wherein the DNA molecule changes its directionality upon entering the nanopore during the ping-pong process. Flipping occurs only on the cis side, suggesting that DNA diffusion is hindered on the trans side. The complex conformations of DNA inside this confined space will be the subject of a future study, as we foresee that ping-pong will be a powerful tool to illuminate the intricate structural dynamics of DNA polymers during multiple recapture processes. Moreover, the ping-pong method can be employed to bind different types of molecules, such as proteins, to the carrier, allowing for the repeated detection of various biomolecules. This versatility enables its potential application in sensing a wide range of targets.

## Supporting Information Available

Detailed materials and methods for molecular ping-pong and supplementary tables and figures of the experimental results (PDF)
