# Supplementary material for: DNA Carrier-Assisted Molecular Ping-Pong in an Asymmetric Nanopore
Source: Nano Lett. 2023 Nov 30;23(23):11145–51. doi: 10.1021/acs.nanolett.3c03605 (PMC10722531; doi:10.1021/acs.nanolett.3c03605)
Supplement: Supplementary file 1 — nl3c03605_si_001.pdf [file nl3c03605_si_001.pdf]

Supporting Information

DNA carrier-assisted molecular ping-pong in an asymmetric nanopore

Fei Zheng[1,3], Mohammed Alawami[1], Jinbo Zhu[1,4], Casey M. Platnich[1], Jingjie Sha[3], Ulrich F. Keyser[1*], and Kaikai Chen[1,2*]

1. Cavendish Laboratory, University of Cambridge, CB3 0HE, Cambridge, United Kingdom

2. School of Nanoscience and Nanotechnology, University of Chinese Academy of Sciences, Beijing, 101408, China

3. Jiangsu Key Laboratory for Design and Manufacture of Micro-Nano Biomedical Instruments, School of Mechanical Engineering, Southeast University, Nanjing, 211100, China

4. School of Biomedical Engineering, Faculty of Medicine, Dalian University of Technology, Dalian, 116024, China

Section 1: Materials and Methods for molecular ping-pong

Nanopore measurement

Glass nanopipettes with diameters of 14 ± 3 nm were fabricated by pulling quartz capillaries (outer diameter = 0.5 mm and inner diameter = 0.2 mm, Sutter Instrument, Heat = 460, Filament = , Velocity = 25, Delay = 170, Pull = 200) using a laser-heating pipette puller (P-2000, Sutter Instrument). Then, the fabricated nanopipettes were assembled into a homemade PDMS chip. The DNA samples were diluted in 4 M LiCl, 10 mM Tris (pH=8.0) to a final concentration of 20 pM. This solution was then added to the cis side of the nanopore. Pure 4 M LiCl solution was added to the trans side. An Axon clamp-patch 200B amplifier (Molecular Devices) was used to apply voltages to drive the DNA through the nanopore and measure the ionic current signal. The signal was filtered with an external Bessel filter (Frequency Devices) at 50 kHz and digitized at a 250 kHz sampling rate with a data card (PCI-6251, National Instruments).

Ping-pong setup

Voltage polarity switching was performed using homemade LabVIEW algorithms. As depicted in **Figure S12**, the LabVIEW script initially reads the current trace through cyclic loops. With a sampling frequency of 250 kHz and 1000 data points per loop, each loop has a duration of 4 ms. The script computes the max-min value (the difference between the maximum and minimum current) for each loop. If the max-min value is below 0.1 nA, it indicates the absence of a blockade. A max-min value between 0.1 nA and 3 nA signifies a blockade event. When the T_{delay} is set at 20 ms, the script initiates a waiting period in the absence of voltage modification. The number of loops required for this waiting period is determined by dividing $T_{delay}$by the duration of each loop (4 ms in this case). Following this waiting period, the script proceeds to reverse the voltage. For example, as shown in **Figure S12b**, upon detecting a blockade event in loop 1, the script begins waiting for the duration of T_{delay}. Loops 2 to 6 represent a waiting phase, consisting of five loops lasting 20 ms. Subsequently, the script initiates the voltage reversal. To achieve less T_{delay}, we changed the data points from 1000 points to 750 and 500, corresponding to 3 ms and 2 ms, respectively. For short DNA polymers like the DNA marker and 8 kb fragment, we used the same T_{delay} for forward and backward translocations (2 ms, 3 ms, 6 ms, 12 ms and 20 ms). For longer ones like lambda DNA, because the backward translocation velocity is substantially slowed down, we set T_{delay} to 20 ms for forward translocations and 60 ms for backward translocations. This is to make sure that next

forward translocation will not appear on the current relaxation part and no trapping of the molecule occurs inside the nanopore. The ping-pong data was extracted and analyzed by a homemade python script.

The linkage of lambda and marker

Lambda DNA has two sticky ends (overhangs) that are complementary to each other. Hence, they may exist in a circular state. First, we heated the lambda sample at constant 70 °C for 10 mins to break the linked sticky ends. Then, we mixed the lambda DNA (1 nM) with linker oligos (3 nM), 4 µl $MgCl_2$, 4 µl 10x Tris, in a tube, and then heated to 85 °C for 5 mins, following a 1 °C every 1 min cooling to 4 °C. Meanwhile, we mixed the single-strand M13mp18 with 189 oligonucleotides (No.2 ~ No.190) without the first one in another tube. Note that if adding 6×8 dumbbells to the carrier, the corresponding staples need to be replaced. The M13mp18 mixture will be heated to 70 °C and then treated with a linear cooling. Finally, we mixed the products in the two tubes and did a room-temperature incubation overnight. To improve the efficiency of hybridisation, we used wide pipettes to transfer the lambda sample and avoided any centrifugation, which may break lambda into fragments. The sequence of the lambda sticky ends and the link oligo are shown in **Table S3**.

Section 2: Supplementary Tables and Figures

Table S1. Sequences of the 190 oligonucleotides complementary to the scaffold.

Oligo No.	Sequence (5' to 3')	Oligo No.	Sequence (5' to 3')
1	TTTTCGTAATCATGGTCATAGCTGTTTCCTGTGTGAAATTGTTATC	96	CTTGAGCCATTTGGGAATTAGAGCCAGCAAAATCACCA
2	CGCTCACAATTCCACACAACATACGAGCCGGAAGCATA	97	GTAGCACCATTACCATTAGCAAGGCCGGAAACGTCACC
3	AAGTGTAAAGCCTGGGGTGCCTAATGAGTGAGCTAACT	98	AATGAAACCATCGATAGCAGCACCGTAATCAGTAGCGA
4	CACATTAATTGCGTTGCGCTCACTGCCCGCTTTCCAGT	99	CAGAATCAAGTTTGCCTTTAGCGTCAGACTGTAGCGCG
5	CGGGAAACCTGTCGTGCCAGCTGCATTAATGAATCGGC	100	TTTTCATCGGCATTTTCGGTCATAGCCCCCTTATTAGC
6	CAACGCGCGGGGAGAGGCGGTTTGCGTATTGGGCGCCA	101	GTTTGCCATCTTTTCATAATCAAAATCACCGGAACCAG
7	GGGTGGTTTTTCTTTTCACCAGTGAGACGGGCAACAGC	102	AGCCACCACCGGAACCGCCTCCCTCAGAGCCGCCACCC
8	TGATTGCCCTTCACCGCCTGGCCCTGAGAGAGTTGCAG	103	TCAGAACCGCCACCCTCAGAGCCACCACCCTCAGAGCC
9	CAAGCGGTCCACGCTGGTTTGCCCCAGCAGGCGAAAAT	104	GCCACCAGAACCACCACCAGAGCCGCCGCCAGCATTGA
10	CCTGTTTGATGGTGGTTCCGAAATCGGCAAAATCCCTT	105	CAGGAGGTTGAGGCAGGTCAGACGATTGGCCTTGATAT
11	ATAAATCAAAAGAATAGCCCGAGATAGGGTTGAGTGTT	106	TCACAAACAAATAAATCCTCATTAAAGCCAGAATGGAA
12	GTTCCAGTTTGGAACAAGAGTCCACTATTAAAGAACGT	107	AGCGCAGTCTCTGAATTTACCGTTCCAGTAAGCGTCAT
13	GGACTCCAACGTCAAAGGGCGAAAAACCGTCTATCAGG	108	ACATGGCTTTTGATGATACAGGAGTGTACTGGTAATAA
14	GCGATGGCCCACTACGTGAACCATCACCCAAATCAAGT	109	GTTTTAACGGGGTCAGTGCCTTGAGTAACAGTGCCCGT
15	TTTTTGGGGTCGAGGTGCCGTAAAGCACTAAATCGGAA	110	ATAAACAGTTAATGCCCCCTGCCTATTTCGGAACCTAT
16	CCCTAAAGGGAGCCCCCGATTTAGAGCTTGACGGGGAA	111	TATTCTGAAACATGAAAGTATTAAGAGGCTGAGACTCC
17	AGCCGGCGAACGTGGCGAGAAAGGAAGGGAAGAAAGCG	112	TCAAGAGAAGGATTAGGATTAGCGGGGTTTTGCTCAGT
18	AAAGGAGCGGGCGCTAGGGCGCTGGCAAGTGTAGCGGT	113	ACCAGGCGGATAAGTGCCGTCGAGAGGGTTGATATAAG
19	CACGCTGCGCGTAACCACCACACCCGCCGCGCTTAATG	114	TATAGCCCGGAATAGGTGTATCACCGTACTCAGGAGGT
20	CGCCGCTACAGGGCGCGTACTATGGTTGCTTTGACGAG	115	TTAGTACCGCCACCCTCAGAACCGCCACCCTCAGAACC
21	CACGTATAACGTGCTTTCCTCGTTAGAATCAGAGCGGG	116	GCCACCCTCAGAGCCACCACCCTCATTTTCAGGGATAG
22	AGCTAAACAGGAGGCCGATTAAAGGGATTTTAGACAGG	117	CAAGCCCAATAGGAACCCATGTACCGTAACACTGAGTT
23	AACGGTACGCCAGAATCCTGAGAAGTGTTTTTATAATC	118	TCGTCACCAGTACAAACTACAACGCCTGTAGCATTCCA
24	AGTGAGGCCACCGAGTAAAAGAGTCTGTCCATCACGCA	119	CAGACAGCCCTCATAGTTAGCGTAACGATCTAAAGTTT
25	AATTAACCGTTGTAGCAATACTTCTTTGATTAGTAATA	120	TGTCGTCTTTCCAGACGTTAGTAAATGAATTTTCTGTA
26	ACATCACTTGCCTGAGTAGAAGAACTCAAACTATCGGC	121	TGGGATTTTGCTAAACAACTTTCAACAGTTTCAGCGGA
27	CTTGCTGGTAATATCCAGAACAATATTACCGCCAGCCA	122	GTGAGAATAGAAAGGAACAACTAAAGGAATTGCGAATA
28	TTGCAACAGGAAAAACGCTCATGGAAATACCTACATTT	123	ATAATTTTTTCACGTTGAAAATCTCCAAAAAAAAGGCT
29	TGACGCTCAATCGTCTGAAATGGATTATTTACATTGGC	124	CCAAAAGGAGCCTTTAATTGTATCGGTTTATCAGCTTG
30	AGATTCACCAGTCACACGACCAGTAATAAAAGGGACAT	125	CTTTCGAGGTGAATTTCTTAAACAGCTTGATACCGATA
31	TCTGGCCAACAGAGATAGAACCCTTCTGACCTGAAAGC	126	GTTGCGCCGACAATGACAACAACCATCGCCCACGCATA
32	GTAAGAATACGTGGCACAGACAATATTTTTGAATGGCT	127	ACCGATATATTCGGTCGCTGAGGCTTGCAGGGAGTTAA
33	ATTAGTCTTTAATGCGCGAACTGATAGCCCTAAAACAT	128	AGGCCGCTTTTGCGGGATCGTCACCCTCAGCAGCGAAA
34	CGCCATTAAAAATACCGAACGAACCACCAGCAGAAGAT	129	GACAGCATCGGAACGAGGGTAGCAACGGCTACAGAGGC
35	AAAACAGAGGTGAGGCGGTCAGTATTAACACCGCCTGC	130	TTTGAGGACTAAAGACTTTTTCATGAGGAAGTTTCCAT
36	AACAGTGCCACGCTGAGAGCCAGCAGCAAATGAAAAAT	131	TAAACGGGTAAAATACGTAATGCCACTACGAAGGCACC
37	CTAAAGCATCACCTTGCTGAACCTCAAATATCAAACCC	132	AACCTAAAACGAAAGAGGCAAAAGAATACACTAAAACA
38	TCAATCAATATCTGGTCAGTTGGCAAATCAACAGTTGA	133	CTCATCTTTGACCCCCAGCGATTATACCAAGCGCGAAA
39	AAGGAATTGAGGAAGGTTATCTAAAATATCTTTAGGAG	134	CAAAGTACAACGGAGATTTGTATCATCGCCTGATAAAT
40	CACTAACAACTAATAGATTAGAGCCGTCAATAGATAAT	135	TGTGTCGAAATCCGCGACCTGCTCCATGTTACTTAGCC
41	ACATTTGAGGATTTAGAAGTATTAGACTTTACAAACAA	136	GGAACGAGGCGCAGACGGTCAATCATAAGGGAACCGAA
42	TTCGACAACTCGTATTAAATCCTTTGCCCGAACGTTAT	137	CTGACCAACTTTGAAAGAGGACAGATGAACGGTGTACA
43	TAATTTTAAAAGTTTGAGTAACATTATCATTTTGCGGA	138	GACCAGGCGCATAGGCTGGCTGACCTTCATCAAGAGTA
44	ACAAAGAAACCACCAGAAGGAGCGGAATTATCATCATA	139	ATCTTGACAAGAACCGGATATTCATTACCCAAATCAAC
45	TTCCTGATTATCAGATGATGGCAATTCATCAATATAAT	140	GTAACAAAGCTGCTCATTCAGTGAATAAGGCTTGCCCT
46	CCTGATTGTTTGGATTATACTTCTGAATAATGGAAGGG	141	GACGAGAAACACCAGAACGAGTAGTAAATTGGGCTTGA
47	TTAGAACCTACCATATCAAAATTATTTGCACGTAAAAC	142	GATGGTTTAATTTCAACTTTAATCATTGTGAATTACCT
48	AGAAATAAAGAAATTGCGTAGATTTTCAGGTTTAACGT	143	TATGCGATTTTAAGAACTGGCTCATTATACCAGTCAGG
49	CAGATGAATATACAGTAACAGTACCTTTTACATCGGGA	144	ACGTTGGGAAGAAAAATCTACGTTAATAAAACGAACTA
50	GAAACAATAACGGATTCGCCTGATTGCTTTGAATACCA	145	ACGGAACAACATTATTACAGGTAGAAAGATTCATCAGT
51	AGTTACAAAATCGCGCAGAGGCGAATTATTCATTTCAA	146	TGAGATTTAGGAATACCACATTCAACTAATGCAGATAC
52	TTACCTGAGCAAAAGAAGATGATGAAACAAACATCAAG	147	ATAACGCCAAAAGGAATTACGAGGCATAGTAAGAGCAA
53	AAAACAAAATTAATTACATTTAACAATTTCATTTGAAT	148	CACTATCATAACCCTCGTTTACCAGACGACGATAAAAA
54	TACCTTTTTTAATGGAAACAGTACATAAATCAATATAT	149	CCAAAATAGCGAGAGGCTTTTGCAAAAGAAGTTTTGCC
55	GTGAGTGAATAACCTTGCTTCTGTAAATCGTCGCTATT	150	AGAGGGGGTAATAGTAAAATGTTTAGACTGGATAGCGT
56	AATTAATTTTCCCTTAGAATCCTTGAAAACATAGCGAT	151	CCAATACTGCGGAATCGTCATAAATATTCATTGAATCC
57	AGCTTAGATTAAGACGCTGAGAAGAGTCAATAGTGAAT	152	CCCTCAAATGCTTTAAACAGTTCAGAAAACGAGAATGA
58	TTATCAAAATCATAGGTCTGAGAGACTACCTTTTTAAC	153	CCATAAATCAAAAATCAGGTCTTTACCCTGACTATTAT
59	CTCCGGCTTAGGTTGGGTTATATAACTATATGTAAATG	154	AGTCAGAAGCAAAGCGGATTGCATCAAAAAGATTAAGA
60	CTGATGCAAATCCAATCGCAAGACAAAGAACGCGAGAA	155	GGAAGCCCGAAAGACTTCAAATATCGCGTTTTAATTCG
61	AACTTTTTCAAATATATTTTAGTTAATTTCATCTTCTG	156	AGCTTCAAAGCGAACCAGACCGGAAGCAAACTCCAACA

62	ACCTAAATTTAATGGTTTGAAATACCGACCGTGTGATA	157	GGTCAGGATTAGAGAGTACCTTTAATTGCTCCTTTTGA
63	AATAAGGCGTTAAATAAGAATAAACACCGGAATCATAA	158	TAAGAGGTCATTTTTGCGGATGGCTTAGAGCTTAATTG
64	TTACTAGAAAAAGCCTGTTTAGTATCATATGCGTTATA	159	CTGAATATAATGCTGTAGCTCAACATGTTTTAAATATG
65	CAAATTCTTACCAGTATAAAGCCAACGCTCAACAGTAG	160	CAACTAAAGTACGGTGTCTGGAAGTTTCATTCCATATA
66	GGCTTAATTGAGAATCGCCATATTTAACAACGCCAACA	161	ACAGTTGATTCCCAATTCTGCGAACGAGTAGATTTAGT
67	TGTAATTTAGGCAGAGGCATTTTCGAGCCAGTAATAAG	162	TTGACCATTAGATACATTTCGCAAATGGTCAATAACCT
68	AGAATATAAAGTACCGACAAAAGGTAAAGTAATTCTGT	163	GTTTAGCTATATTTTCATTTGGGGCGCGAGCTGAAAAG
69	CCAGACGACGACAATAAACAACATGTTCAGCTAATGCA	164	GTGGCATCAATTCTACTAATAGTAGTAGCATTAACATC
70	GAACGCGCCTGTTTATCAACAATAGATAAGTCCTGAAC	165	CAATAAATCATACAGGCAAGGCAAAGAATTAGCAAAAT
71	AAGAAAAATAATATCCCATCCTAATTTACGAGCATGTA	166	TAAGCAATAAAGCCTCAGAGCATAAAGCTAAATCGGTT
72	GAAACCAATCAATAATCGGCTGTCTTTCCTTATCATTC	167	GTACCAAAAACATTATGACCCTGTAATACTTTTGCGGG
73	CAAGAACGGGTATTAAACCAAGTACCGCACTCATCGAG	168	AGAAGCCTTTATTTCAACGCAAGGATAAAAATTTTTAG
74	AACAAGCAAGCCGTTTTTATTTTCATCGTAGGAATCAT	169	AACCCTCATATATTTTAAATGCAATGCCTGAGTAATGT
75	TACCGCGCCCAATAGCAAGCAAATCAGATATAGAAGGC	170	GTAGGTAAAGATTCAAAAGGGTGAGAAAGGCCGGAGAC
76	TTATCCGGTATTCTAAGAACGCGAGGCGTTTTAGCGAA	171	AGTCAAATCACCATCAATATGATATTCAACCGTTCTAG
77	CCTCCCGACTTGCGGGAGGTTTTGAAGCCTTAAATCAA	172	CTGATAAATTAATGCCGGAGAGGGTAGCTATTTTTGAG
78	GATTAGTTGCTATTTTGCACCCAGCTACAATTTTATCC	173	AGATCTACAAAGGCTATCAGGTCATTGCCTGAGAGTCT
79	TGAATCTTACCAACGCTAACGAGCGTCTTTCCAGAGCC	174	GGAGCAAACAAGAGAATCGATGAACGGTAATCGTAAAA
80	TAATTTGCCAGTTACAAAATAAACAGCCATATTATTTA	175	CTAGCATGTCAATCATATGTACCCCGGTTGATAATCAG
81	TCCCAATCCAAATAAGAAACGATTTTTTGTTTAACGTC	176	AAAAGCCCCAAAAACAGGAAGATTGTATAAGCAAATAT
82	AAAAATGAAAATAGCAGCCTTTACAGAGAGAATAACAT	177	TTAAATTGTAAACGTTAATATTTTGTTAAAATTCGCAT
83	AAAAACAGGGAAGCGCATTAGACGGGAGAATTAACTGA	178	TAAATTTTTGTTAAATCAGCTCATTTTTTAACCAATAG
84	ACACCCTGAACAAAGTCAGAGGGTAATTGAGCGCTAAT	179	GAACGCCATCAAAAATAATTCGCGTCTGGCCTTCCTGT
85	ATCAGAGAGATAACCCACAAGAATTGAGTTAAGCCCAA	180	AGCCAGCTTTCATCAACATTAAATGTGAGCGAGTAACA
86	TAATAAGAGCAAGAAACAATGAAATAGCAATAGCTATC	181	ACCCGTCGGATTCTCCGTGGGAACAAACGGCGGATTGA
87	TTACCGAAGCCCTTTTTAAGAAAAGTAAGCAGATAGCC	182	CCGTAATGGGATAGGTCACGTTGGTGTAGATGGGCGCA
88	GAACAAAGTTACCAGAAGGAAACCGAGGAAACGCAATA	183	TCGTAACCGTGCATCTGCCAGTTTGAGGGGACGACGAC
89	ATAACGGAATACCCAAAAGAACTGGCATGATTAAGACT	184	AGTATCGGCCTCAGGAAGATCGCACTCCAGCCAGCTTT
90	CCTTATTACGCAGTATGTTAGCAAACGTAGAAAATACA	185	CCGGCACCGCTTCTGGTGCCGGAAACCAGGCAAAGCGC
91	TACATAAAGGTGGCAACATATAAAAGAAACGCAAAGAC	186	CATTCGCCATTCAGGCTGCGCAACTGTTGGGAAGGGCG
92	ACCACGGAATAAGTTTATTTTGTCACAATCAATAGAAA	187	ATCGGTGCGGGCCTCTTCGCTATTACGCCAGCTGGCGA
93	ATTCATATGGTTTACCAGCGCCAAAGACAAAAGGGCGA	188	AAGGGGGATGTGCTGCAAGGCGATTAAGTTGGGTAACG
94	CATTCAACCGATTGAGGGAGGGAAGGTAAATATTGACG	189	CCAGGGTTTTCCCAGTCACGACGTTGTAAAACGACGGC
95	GAAATTATTCATTAAAGGTGAATTATCACCGTCACCGA	190	CAGTGCCAAGCTTGCATGCCTGCAGGTCGACTCTAGAGGA TCTTTT

Table S2. Sequences of the oligonucleotides to attach 6×8 dumbbells to the carrier.

Group of dumbbells	Sequence (5' to 3')	Oligo to replace
A	ACATCACTTGTCCTCTTTTGAGGAACAAGTTTTCTTGTCCTGAGTAGA	26-30
	AGAACTCAAATCCTCTTTTGAGGAACAAGTTTTCTTGTCTATCGGCCT	
	TGCTGGTAATTCCTCTTTTGAGGAACAAGTTTTCTTGTATCCAGAACA	
	ATATTACCGCTCCTCTTTTGAGGAACAAGTTTTCTTGTCAGCCATTGC	
	AACAGGAAAATCCTCTTTTGAGGAACAAGTTTTCTTGTACGCTCATGG	
	AAATACCTACTCCTCTTTTGAGGAACAAGTTTTCTTGTATTTTGACGC	
	TCAATCGTCTTCCTCTTTTGAGGAACAAGTTTTCTTGTGAAATGGATT	
	ATTTACATTGTCCTCTTTTGAGGAACAAGTTTTCTTGTGCAGATTCAC	
	CAGTCACACGACCAGTAATAAAAGGGACAT	
B	TTACCTGAGCAAAAGAAGATGATGAAACAAACATCAAGAAAACA	52-57
	AAATTAATTATCCTCTTTTGAGGAACAAGTTTTCTTGTCATTTAACAA	
	TTTCATTTGATCCTCTTTTGAGGAACAAGTTTTCTTGTATTACCTTTT	
	TTAATGGAAATCCTCTTTTGAGGAACAAGTTTTCTTGTCAGTACATAA	
	ATCAATATATTCCTCTTTTGAGGAACAAGTTTTCTTGTGTGAGTGAAT	
	AACCTTGCTTTCCTCTTTTGAGGAACAAGTTTTCTTGTCTGTAAATCG	
	TCGCTATTAATCCTCTTTTGAGGAACAAGTTTTCTTGTTTAATTTTCC	
	CTTAGAATCCTCCTCTTTTGAGGAACAAGTTTTCTTGTTTGAAAACAT	
	AGCGATAGCTTCCTCTTTTGAGGAACAAGTTTTCTTGTTAGATTAAGA	
	CGCTGAGAAGAGTCAATAGTGAAT	
C	TGAATCTTACCAACGCTAACGAGCGTCTTTCCAGAGCCTAATTTGCCAGT	79-85
	TACAAAATAATCCTCTTTTGAGGAACAAGTTTTCTTGTACAGCCATAT	
	TATTTATCCCTCCTCTTTTGAGGAACAAGTTTTCTTGTAATCCAAATA	
	AGAAACGATTTCCTCTTTTGAGGAACAAGTTTTCTTGTTTTTGTTTAA	
	CGTCAAAAATTCCTCTTTTGAGGAACAAGTTTTCTTGTGAAAATAGCA	
	GCCTTTACAGTCCTCTTTTGAGGAACAAGTTTTCTTGTAGAGAATAAC	
	ATAAAAACAGTCCTCTTTTGAGGAACAAGTTTTCTTGTGGAAGCGCAT	
	TAGACGGGAGTCCTCTTTTGAGGAACAAGTTTTCTTGTAATTAACTGA	
	ACACCCTGAATCCTCTTTTGAGGAACAAGTTTTCTTGTCAAAGTCAGA	
	GGGTAATTGAGCGCTAATATCAGAGAGATAACCCACAAGAATTGAGTTAAGCCCAA	
D	TCACAAACAAATAAATCCTCATTAAAGCCAGAATGGAAAGCGCAGTCTCTGAATTT	106-112
	ACCGTTCCAGTCCTCTTTTGAGGAACAAGTTTTCTTGTTAAGCGTCAT	
	ACATGGCTTTTCCTCTTTTGAGGAACAAGTTTTCTTGTTGATGATACA	
	GGAGTGTACTTCCTCTTTTGAGGAACAAGTTTTCTTGTGGTAATAAGT	
	TTTAACGGGGTCCTCTTTTGAGGAACAAGTTTTCTTGTTCAGTGCCTT	
	GAGTAACAGTTCCTCTTTTGAGGAACAAGTTTTCTTGTGCCCGTATAA	
	ACAGTTAATGTCCTCTTTTGAGGAACAAGTTTTCTTGTCCCCCTGCCT	
	ATTTCGGAACTCCTCTTTTGAGGAACAAGTTTTCTTGTCTATTATTCT	
	GAAACATGAATCCTCTTTTGAGGAACAAGTTTTCTTGTAGTATTAAGA	
	GGCTGAGACTCCTCAAGAGAAGGATTAGGATTAGCGGGGTTTTGCTCAGT	
E	CAAAGTACAACGGAGATTTGTATC	134-139
	ATCGCCTGATTCCTCTTTTGAGGAACAAGTTTTCTTGTAAATTGTGTC	
	GAAATCCGCGTCCTCTTTTGAGGAACAAGTTTTCTTGTACCTGCTCCA	
	TGTTACTTAGTCCTCTTTTGAGGAACAAGTTTTCTTGTCCGGAACGAG	
	GCGCAGACGGTCCTCTTTTGAGGAACAAGTTTTCTTGTTCAATCATAA	
	GGGAACCGAATCCTCTTTTGAGGAACAAGTTTTCTTGTCTGACCAACT	
	TTGAAAGAGGTCCTCTTTTGAGGAACAAGTTTTCTTGTACAGATGAAC	
	GGTGTACAGATCCTCTTTTGAGGAACAAGTTTTCTTGTCCAGGCGCAT	
	AGGCTGGCTGTCCTCTTTTGAGGAACAAGTTTTCTTGTACCTTCATCA	
	AGAGTAATCTTGACAAGAACCGGATATTCATTACCCAAATCAAC	
F	ACAGTTGATTCCCAATTCTGCGAACGAGTA	161-165
	GATTTAGTTTTCCTCTTTTGAGGAACAAGTTTTCTTGTGACCATTAGA	
	TACATTTCGCTCCTCTTTTGAGGAACAAGTTTTCTTGTAAATGGTCAA	
	TAACCTGTTTTCCTCTTTTGAGGAACAAGTTTTCTTGTAGCTATATTT	
	TCATTTGGGGTCCTCTTTTGAGGAACAAGTTTTCTTGTCGCGAGCTGA	
	AAAGGTGGCATCCTCTTTTGAGGAACAAGTTTTCTTGTTCAATTCTAC	
	TAATAGTAGTTCCTCTTTTGAGGAACAAGTTTTCTTGTAGCATTAACA	
	TCCAATAAATTCCTCTTTTGAGGAACAAGTTTTCTTGTCATACAGGCA	
	AGGCAAAGAATCCTCTTTTGAGGAACAAGTTTTCTTGTTTAGCAAAAT	

Table S2. Sequences of the oligonucleotides to attach 6×8 dumbbells to the carrier.

Table S3. Sequences of the sticky ends and oligonucleotide to link the marker to the lambda carrier. The sequence marked in red is complementary to the 5' sticky end and that marked in purple is complementary to the 3' overhang of the marker without the first oligo.

Cohesive ends & linker	sequence(5' to 3')
Lambda cohesive end #1	5' GGGCGGCGACCT 3'
Lambda cohesive end #2	5' AGGTCGCCGCCC 3'
Linker oligo	5'AGGTCGCCGCCCGTAATCATGGTCATAGCTGTTTCCTGTGTGAAATTGTTATC 3'

Table S4. Number of each recapture cycle of different delay times, τ_{delay} for 8 kb DNA. For each τ_{delay}, we recorded the current traces for 1h and then counted the number of each recapture cycle, thereby obtaining the percentage of each recapture cycle from dividing by total number of ping-pong process.

τ_{delay} (ms)	**Number of each recapture cycle**									
	**1**	**2**	**3**	**4**	**5**	**6**	**7**	**8**	**9**	**10**
20	169 (69%)	48 (20%)	18 (7%)	5 (2%)	1 (0.4%)	2 (0.8%)	2 (0.8%)	0	0	0
12	161 (64%)	54 (22%)	14 (6%)	13 (5%)	5 (2%)	1 (0.4%)	2 (0.8%)	1 (0.4%)	0	0
6	199 (59%)	76 (22%)	35 (10%)	19 (6%)	7 (2%)	0	1 (0.3%)	1 (0.3%)	0	0
3	119 (55%)	41 (19%)	26 (12%)	16 (7%)	5 (2%)	4 (2%)	0	3 (1%)	1 (0.5%)	0
2	109 (60%)	26 (15%)	19 (11%)	11 (6%)	5 (3%)	2 (1%)	3 (2%)	1 (0.5%)	2 (1%)	1 (0.5%)

Table S5. Number of each recapture cycle of different negative voltages for 8 kb DNA. τ_{delay} was 3 ms. The positive voltage was all set at +600 mV. We expected the molecule would have a lower velocity under a lower voltage in the backward translocation towards the cis side, then it could be recaptured more times. However, we did not observe an obvious improvement from the result.

**Negative voltage**	**Number of each recapture cycle**									
	**1**	**2**	**3**	**4**	**5**	**6**	**7**	**8**	**9**	**10**
-600 mV	131 (52%)	73 (29%)	23 (9%)	12 (5%)	6 (2%)	5 (2%)	1 (0.4%)	0	0	0
-400 mV	175 (61%)	73 (26%)	20 (7%)	8 (3%)	5 (2%)	2 (0.7%)	2 (0.7%)	0	0	0

Table S6. Number of each recapture cycle of different pore sizes for 8 kb DNA. τ_{delay} was 3 ms. The pore sizes were evaluated by the current baseline at +600 mV. We expect that a larger pore could potentially facilitate more molecule recaptures, considering that the capture area of the pore is proportional to the square of the pore diameter. However, while a larger pore did lead to a reduction in the percentage of one recapture cycle and an increase in the percentage of two recapture cycles, it did not significantly boost the percentage of multiple recapture cycles (five or more recapture cycles).

**Pore size**	**Number of each recapture cycle**									
	**1**	**2**	**3**	**4**	**5**	**6**	**7**	**8**	**9**	**10**
8 nA	133 (73%)	25 (14%)	12 (7%)	7 (4%)	3 (2%)	1 (0.5%)	0	1 (0.5%)	0	0
10 nA	119 (55%)	41 (19%)	26 (12%)	16 (7%)	5 (2%)	4 (2%)	0	3 (1%)	1 (0.5%)	0
16 nA	131 (52%)	73 (29%)	23 (9%)	12 (5%)	6 (2%)	5 (2%)	1 (0.4%)	0	0	0

Table S7. Number of each recapture cycle of three pores with the same size for 48.5 kb lambda DNA. τ_{delay} was 20 ms for the forward translocation and 60 ms for the backward translocation. The current baselines of the 3 pores at 600 mV were 9.97, 10.45, and 8.95 nA, respectively.

**Pore index**	**Number of each recapture cycle**									
Pore #1	**1**	**2**	**3**	**4**	**5**	**6**	**7**	**8**	**9**	**10**
	43 (25%)	25 (15%)	23 (13%)	18 (10%)	7 (4%)	13 (8%)	4 (2%)	8 (5%)	2 (1%)	4 (2%)
	**11**	**12**	**13**	**14**	**15**	**16**	**17**	**18**	**19**	**20**
	3 (2%)	4 (2%)	2 (1%)	3 (2%)	2 (1%)	0	2 (1%)	3 (2%)	0	2 (1%)
	**21**	**23**	**30**	**55**	**Total**					
	1 (0.6%)	1 (0.6%)	1 (0.6%)	1 (0.6%)	172					
Pore #2	**1**	**2**	**3**	**4**	**5**	**6**	**7**	**8**	**9**	**10**
	45 (28%)	25 (16%)	17 (11%)	14 (9%)	11 (7%)	7 (4%)	9 (6%)	6 (4%)	7 (4%)	4 (2%)
	**11**	**12**	**13**	**14**	**15**	**16**	**17**	**18**	**19**	**20**
	0	2 (1%)	3 (2%)	3 (2%)	1 (0.6%)	0	2 (1%)	0	3 (2%)	1 (0.6%)
	**22**	**24**	**25**	**27**	**31**	**Total**				
	1 (0.6%)	1 (0.6%)	1 (0.6%)	1 (0.6%)	1 (0.6%)	161				
Pore #3	**1**	**2**	**3**	**4**	**5**	**6**	**7**	**8**	**9**	**10**
	32 (26%)	20 (17%)	7 (6%)	14 (12%)	9 (7%)	7 (6%)	4 (3%)	8 (7%)	1 (0.8%)	2 (2%)
	**11**	**12**	**13**	**14**	**15**	**16**	**17**	**18**	**19**	**20**
	0	1 (0.8%)	2 (2%)	2 (2%)	3 (2%)	0	1 (0.8%)	0	3 (2%)	1 (0.8%)
	**21**	**24**	**30**	**57**	**Total**					
	1 (0.8%)	1 (0.8%)	1 (0.8%)	1 (0.8%)	121					

Table S8. The types of DNA molecules used in this work and its modification state.

DNA type	Length	Modification
7.2 kb M13mp18	7.2 kb	6x8 dumbbells
8 kb	8 kb	no modification
Lambda DNA	48.5 kb	no modification
Lambda + marker DNA	55.7 kb	6x8 dumbbells

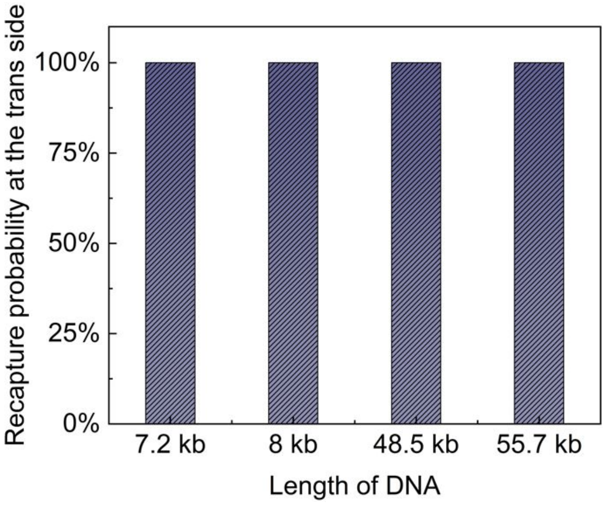

Figure S1. Recapture probability at the trans side for different lengths of DNA molecules (7.2 kb, 8 kb, 48.5 kb, 55.7 kb). Each probability was derived from the analysis of above 1000 ping-pong events. The probabilities were all 100%, showing that the nanoconfinement at the trans side did restrict the diffusion of the DNA molecule strongly. The detail of different types of DNA molecules used here can be found in **Table S8**.

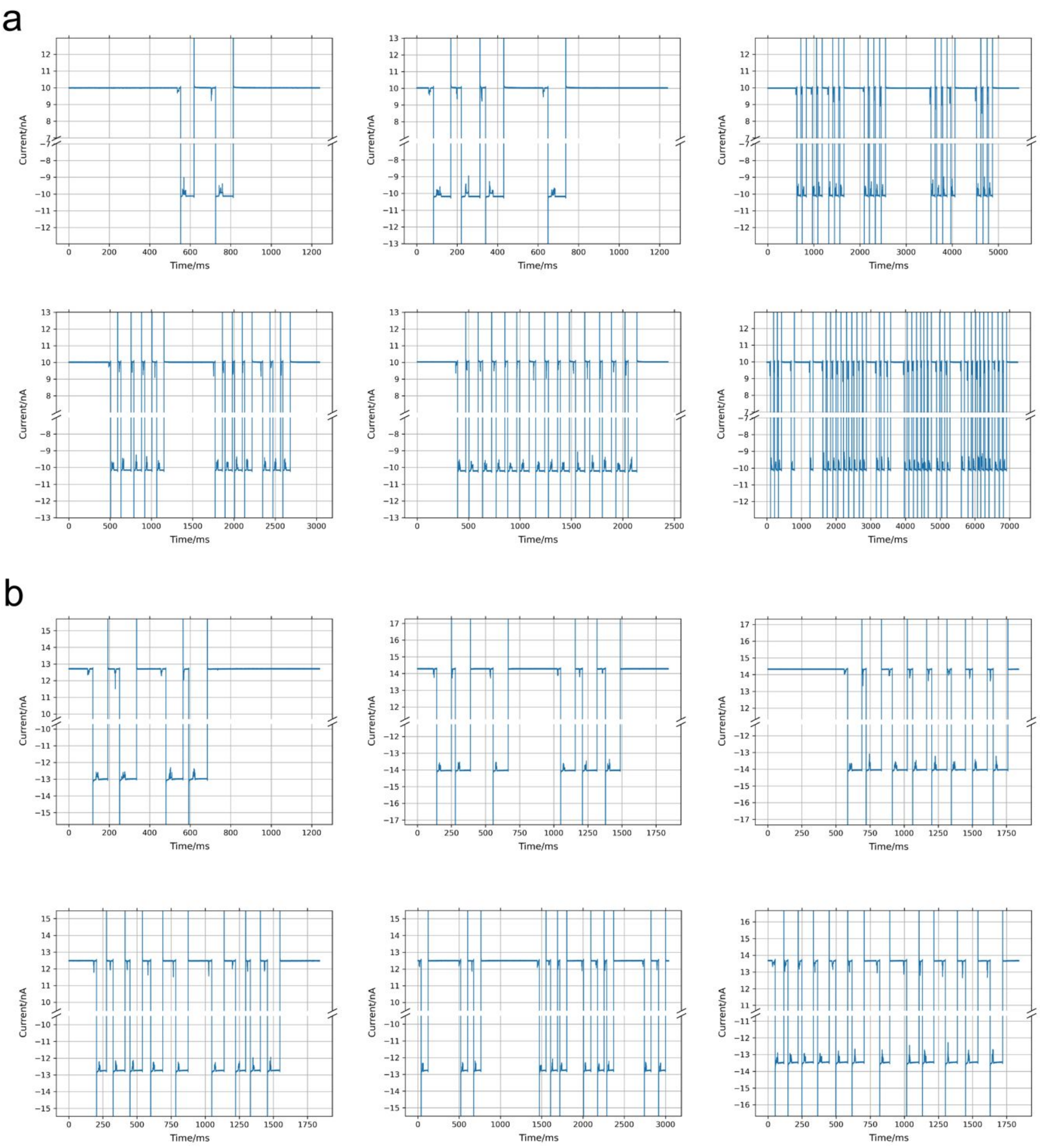

Figure S2. Examples of ping-pong process of (a) 48.5 kb lambda DNA, (b)55.7 kb lambda + marker construct. The molecule was always recaptured in the backward (trans-cis) direction and lost in the forward (cis-trans) direction.

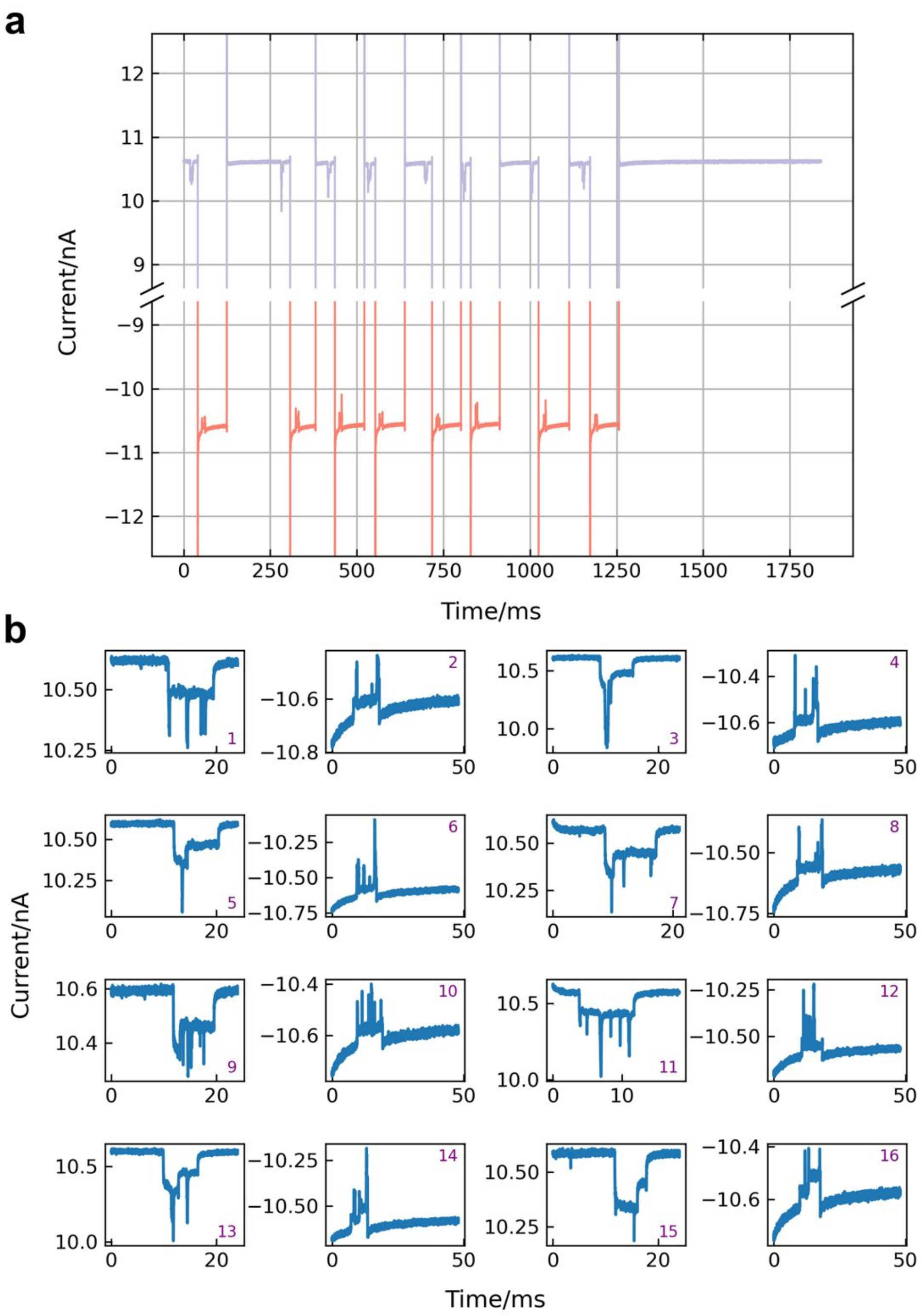

Figure S3. Example of ping-pong in a nanopore under a concentration gradient (cis: 4M trans: 2M). The sample was lambda DNA. a. Overview of the current trace of the ping-pong process; b. Enlarged view of each capture and recapture event. We expected that a concentration gradient would generate a diffusion-osmosis flow (from cis side to trans side) that hindered the backward translocation of the DNA molecule, thereby increasing recaptures. However, this led to another problem that the current recovery to the baseline after the voltage reverse required more time for a lower concentration. This issue was more severe for the backward direction. This will cause difficulties in the signal analysis.

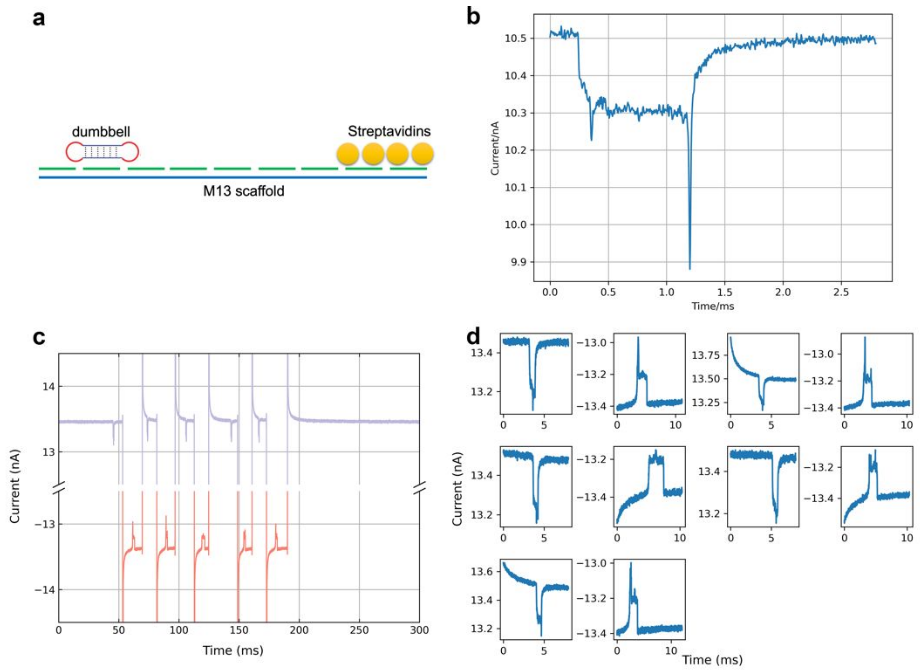

Figure S4. Example of ping-pong for the marker bound with 4 streptavidin. a. Design of the structures on the M13mp18 scaffold. A dumbbell structure was designed at the beginning of the scaffold and 4 streptavidin were arranged at the end; b. A typical translocation signal of the structural molecule. The signal consisted of a small spike at the beginning (dumbbell) and a large spike at the end (4 streptavidins); c. The maximum number of recapture cycles (5) we could achieve from this structured molecule; d. Enlarged view of each capture and recapture event. We expected that the binding of large streptavidins (5 nm globular diameter, 60 kDa) may slow down the translocation of the DNA molecule, hence we could obtain a greater number of recaptures by this way. The structure of 4 streptavidins did generate a large spike signal. However, the maximum number of recapture cycles we could get was 5 times, which was same as that of no-streptavidin DNA. We attributed this to that only 4 streptavidins (60 kDa × 4) cannot make a difference to the diffusion of the entire molecule.

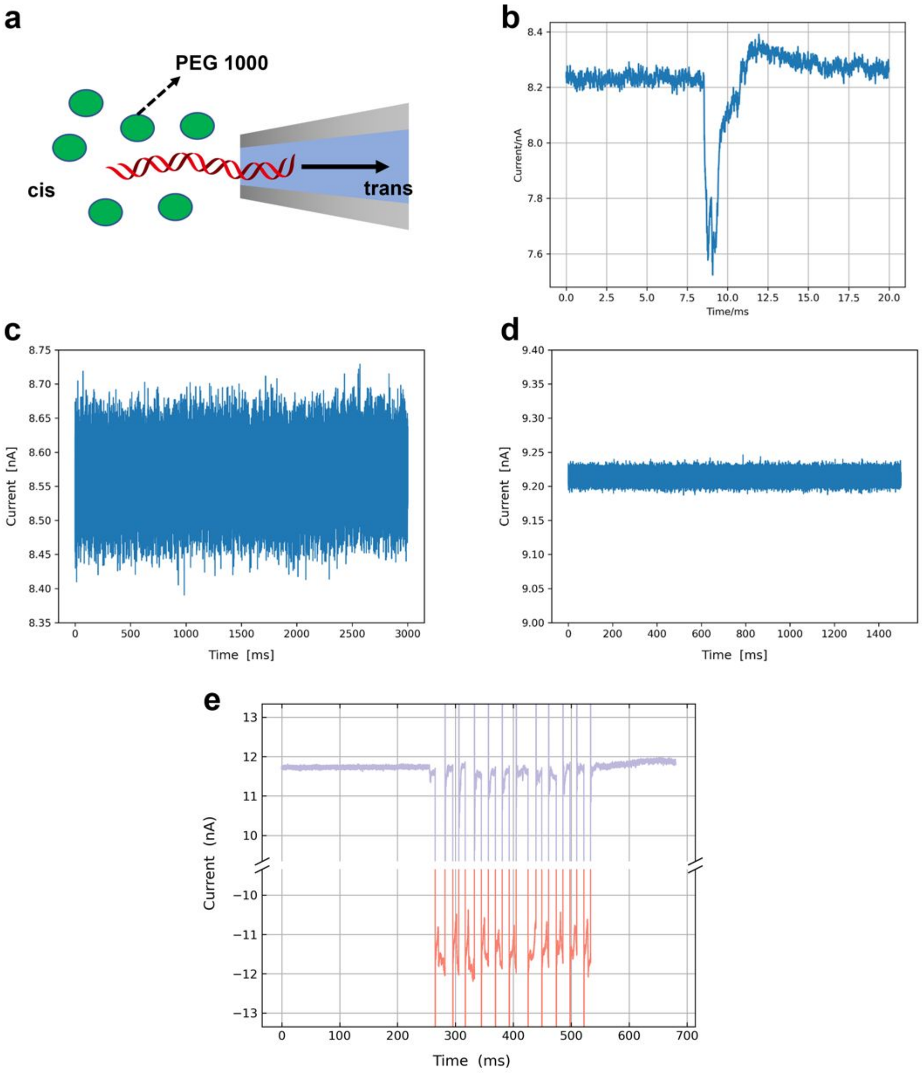

Figure S5. Example of ping-pong in a macromolecule-electrolyte solution. The solution was 10% PEG1000 w/v + 4M LiCl. The sample was 8k DNA. a. Schematic of the macromolecule experiment. The cis side was filled with 10% PEG1000 w/v + 4M LiCl and the trans side was filled with pure 4M LiCl; b. A typical translocation signal of 8k DNA molecule in this solution; c. The current trace of 10% PEG8000 w/v + 4M LiCl; d. The current trace of 4M LiCl; e. An example of ping-pong process in this scenario. We expected that the big-sized macromolecules (PEG1000) at the cis side would have a steric hindrance for the backward-translocating DNA molecule and slow down its motion. However, the solution with macromolecules significantly increased the noise[1], which would make it difficult to identify nanostructures on the DNA scaffold. Additionally, the DNA molecule was easily trapped inside the nanopore during the ping-pong process.

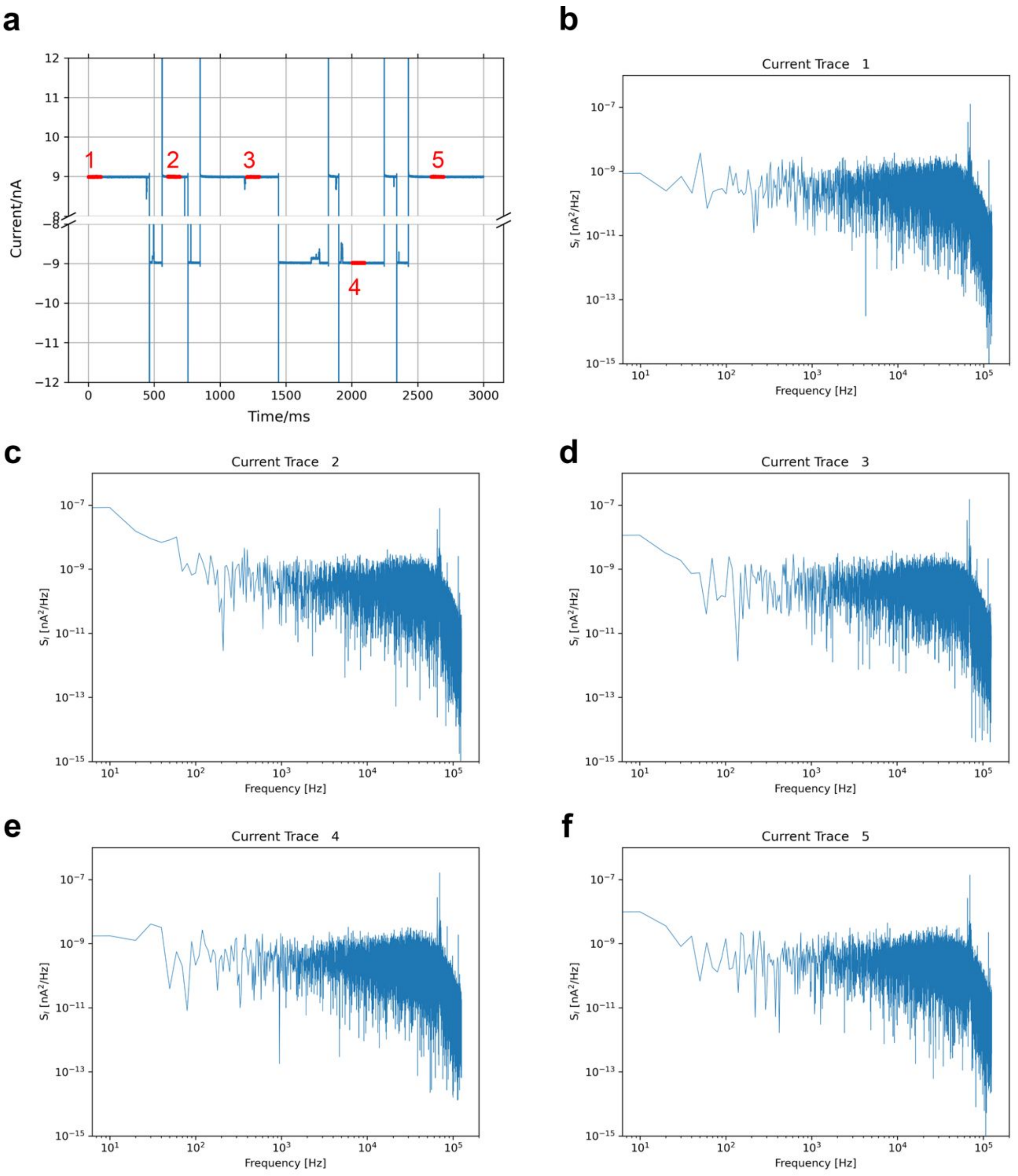

Figure S6. Power density spectrum (PSD) of the current trace before, within, and after ping-pong. a. The entire current trace of a ping-pong process. b-f. PSD of the current trace 1-5. We used fft to transform the current trace of ping-pong to PSD and investigated whether the DNA recapture would influence the noise distribution, i.e., whether there was a strong interaction between the DNA molecule and the nanopore surface. Results indicated that the current noise was not affected by ping-pong.

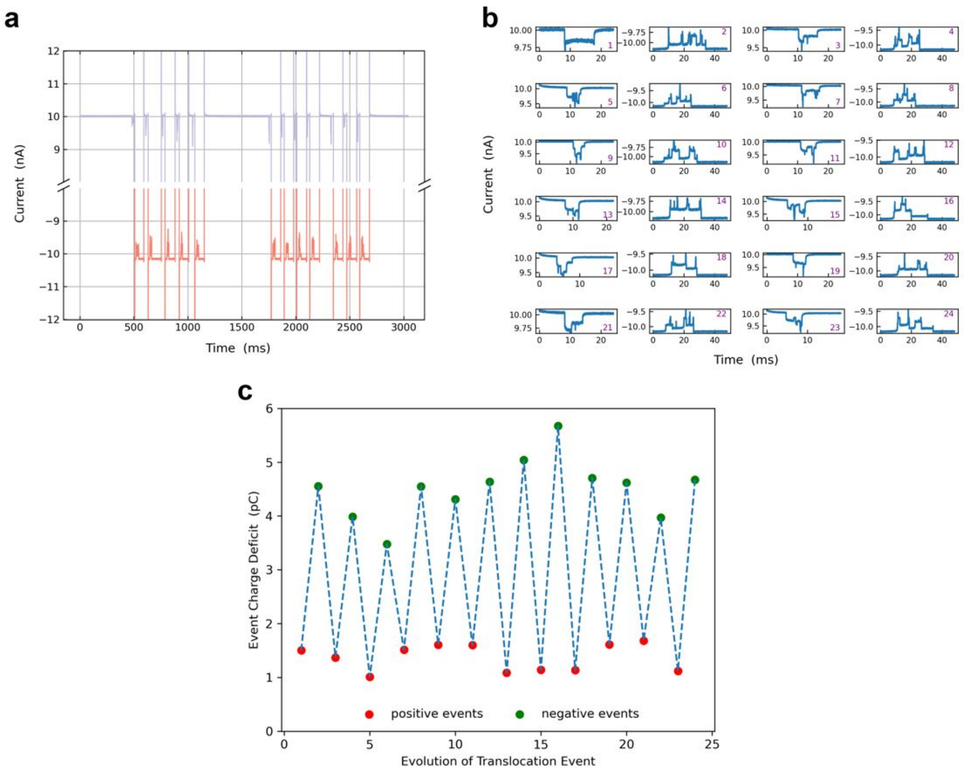

Figure S7. Event charge deficit (ECD) evolution during ping-pong process. The sample was lambda DNA. a. Example of a ping-pong process; b. Enlarged view of each capture and recapture event; c. ECD value as a function of translocation event index. We also investigated the ECD profile during the ping-pong process. ECD values almost were the same for both forward translocation events and backward ones, indicating that these events were full translocations.

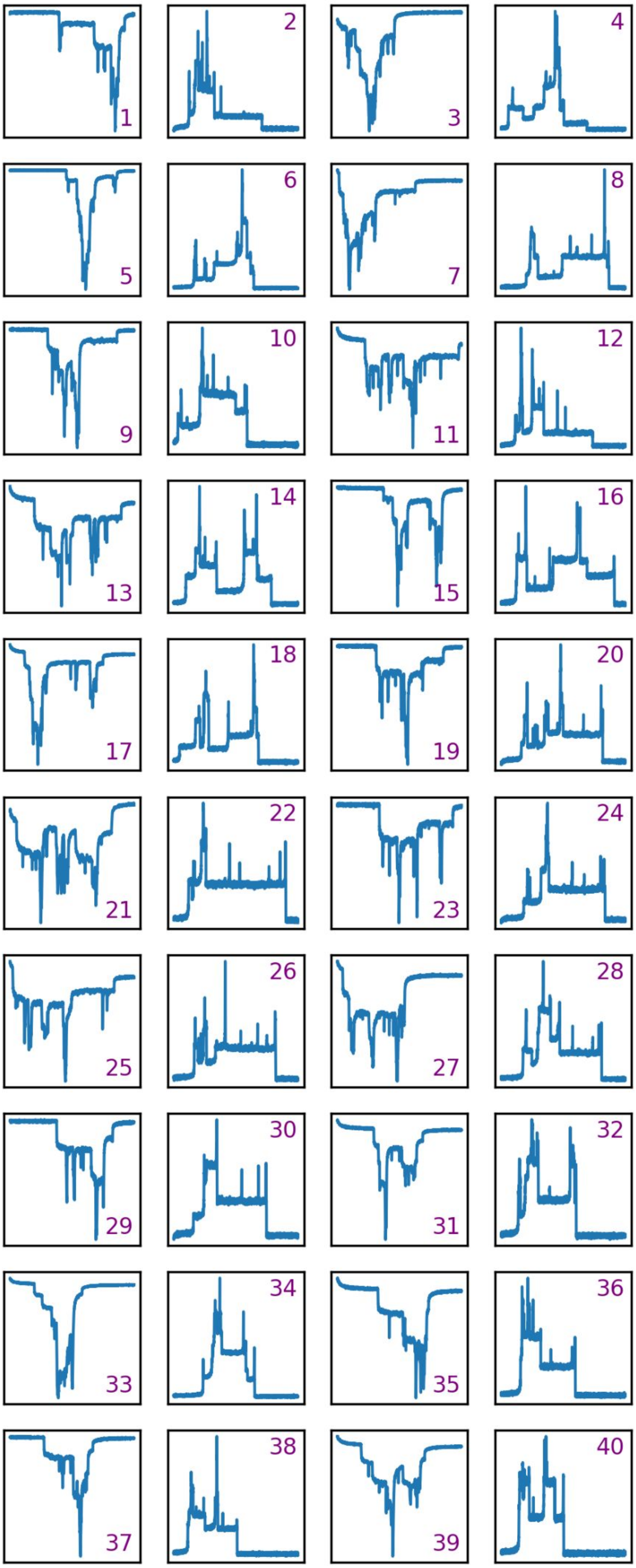
1
2
3
4
5
6
7
8
9
10
11
12
13
14
15
16
17
18
19
20
21
22
23
24
25
26
27
28
29
30
31
32
33
34
35
36
37
38
39
40

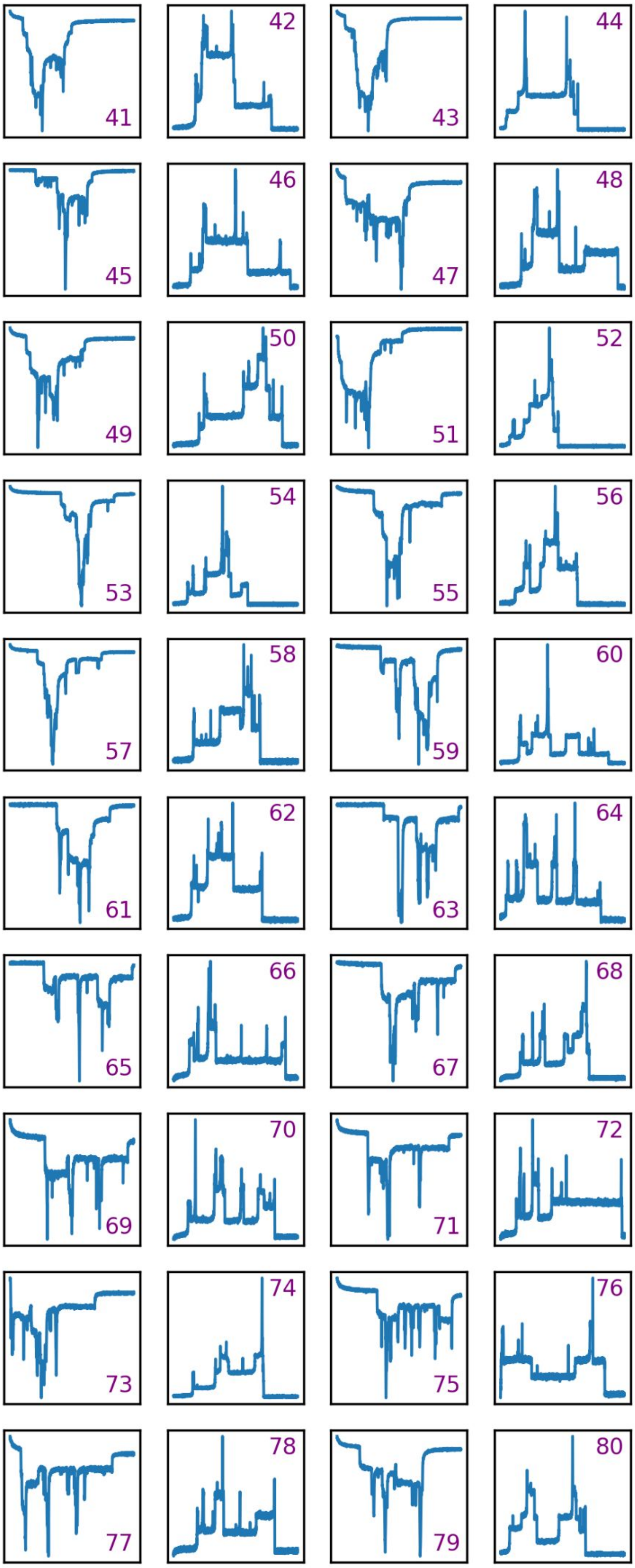
41
42
43
44
45
46
47
48
49
50
51
52
53
54
55
56
57
58
59
60
61
62
63
64
65
66
67
68
69
70
71
72
73
74
75
76
77
78
79
80

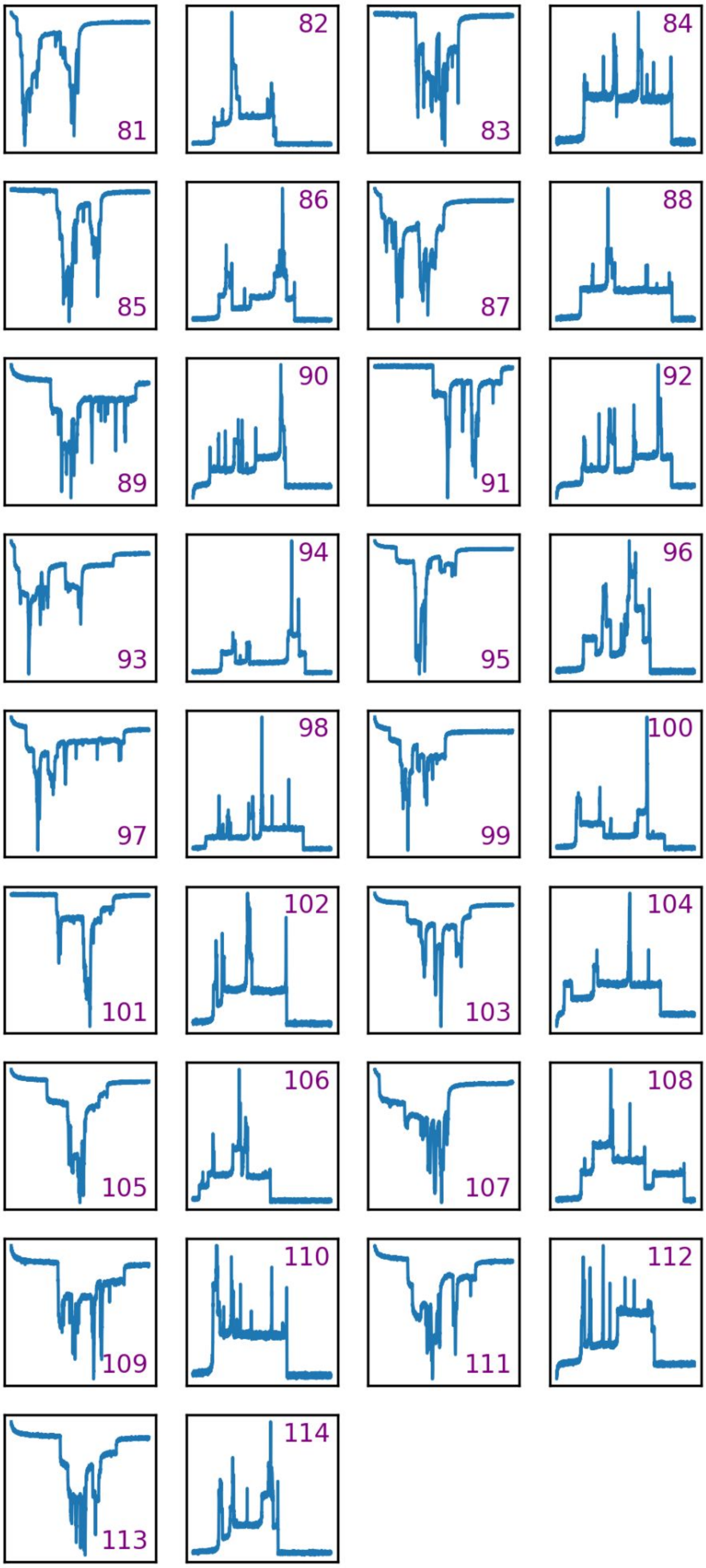

Figure S8. Enlarged view of each capture and recapture events in the 57-cycle (114 captures) recapture ping-pong process. Note that, the axis ranges of the events are not the same.

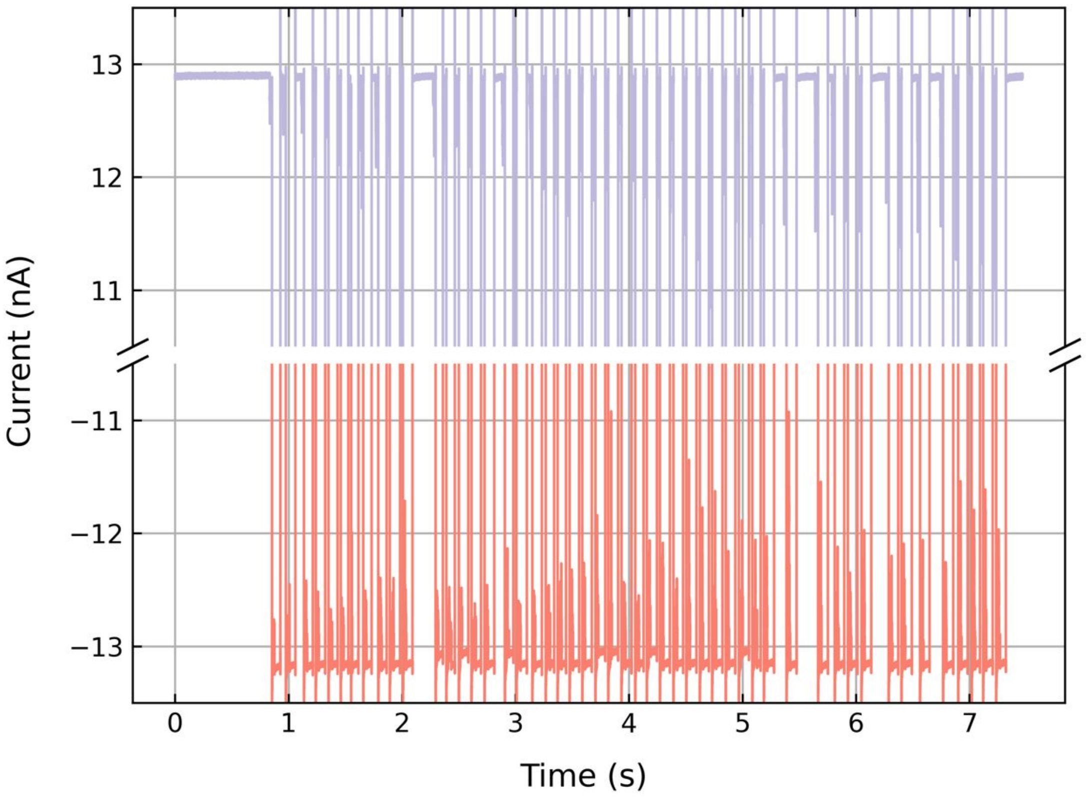

Figure S9. Example of a 50-cycle (100 captures) recapture ping-pong for the lambda + marker construct.

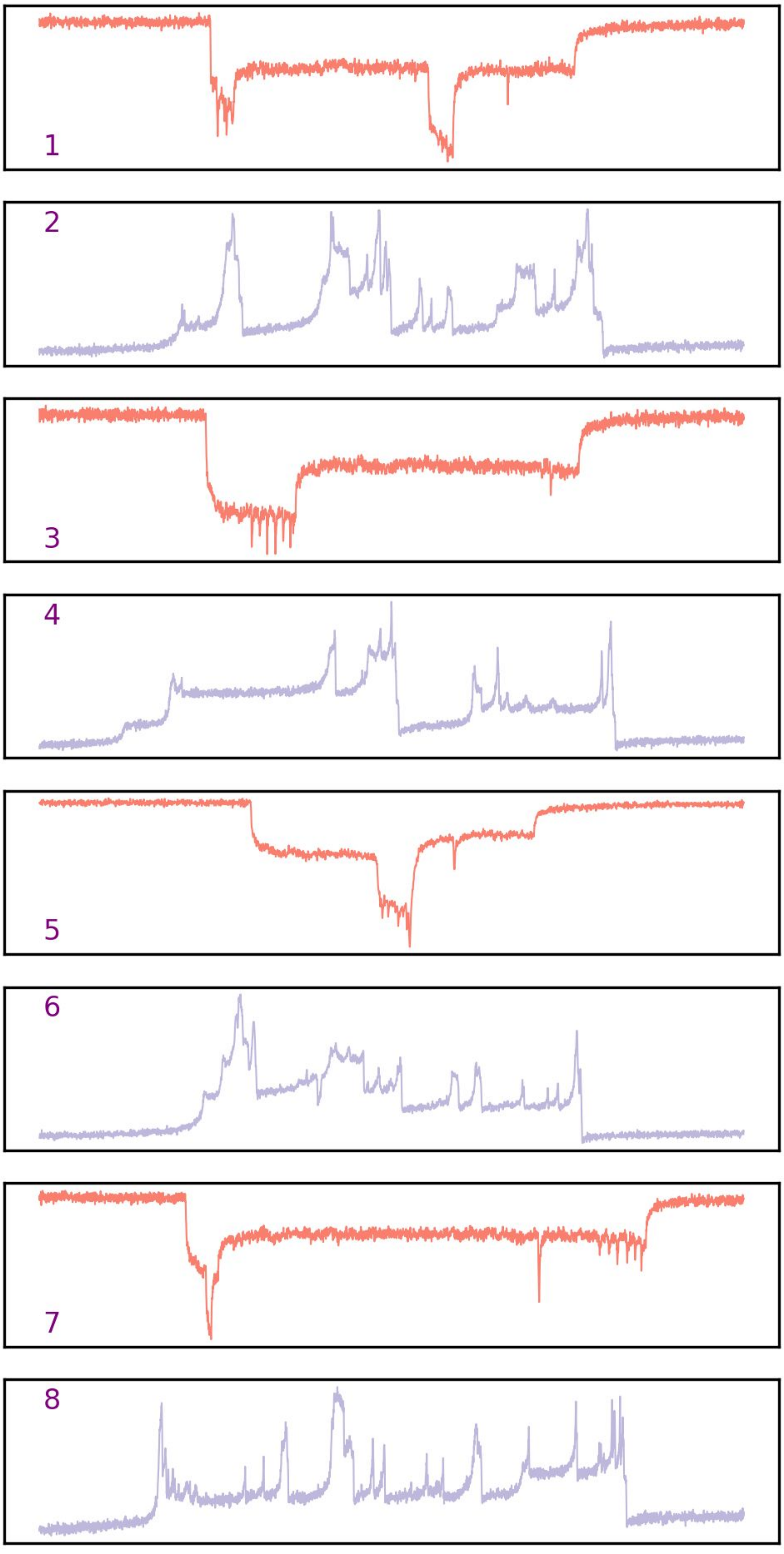
1
2
3
4
5
6
7
8

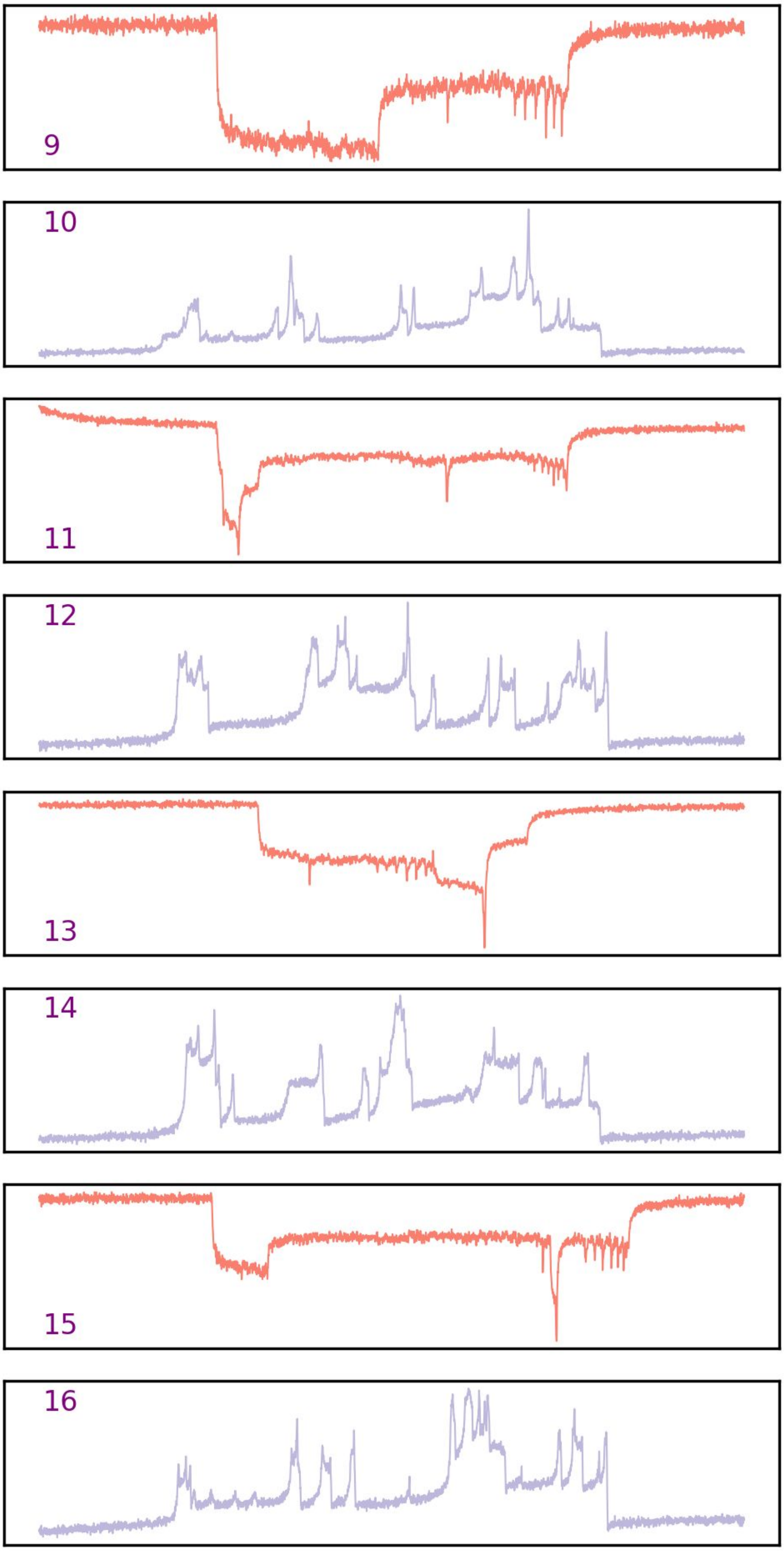
9
10
11
12
13
14
15
16

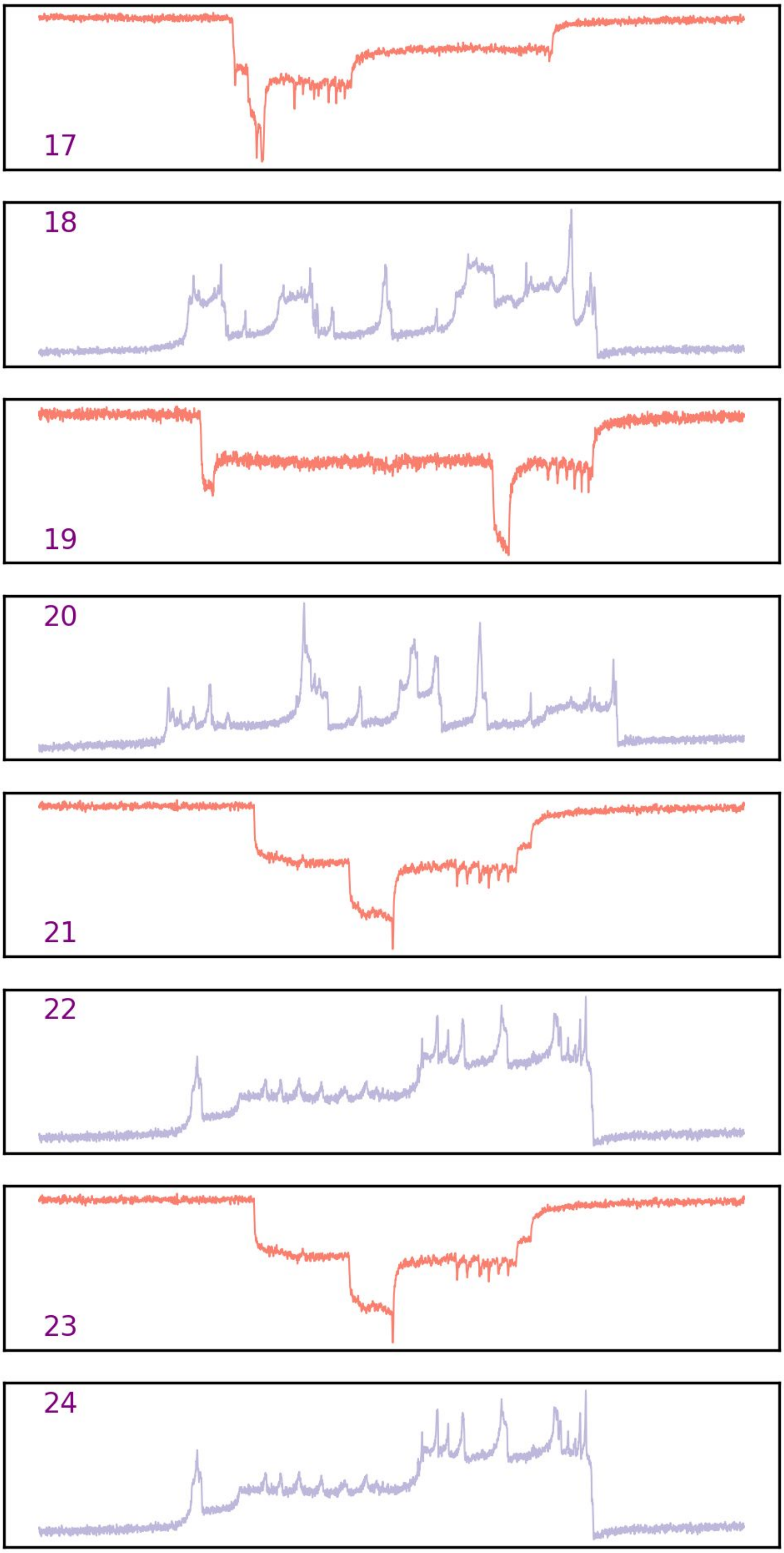
17
18
19
20
21
22
23
24

Figure S10. Examples of the enlarged view of recapture events in a 11-cycle recapture ping-pong for the lambda + marker construct with froward translocations shown in red and backward translocations shown in purple. Note that, to have a better view at the signals of the nanostructure, the axis ranges of the events are not the same.

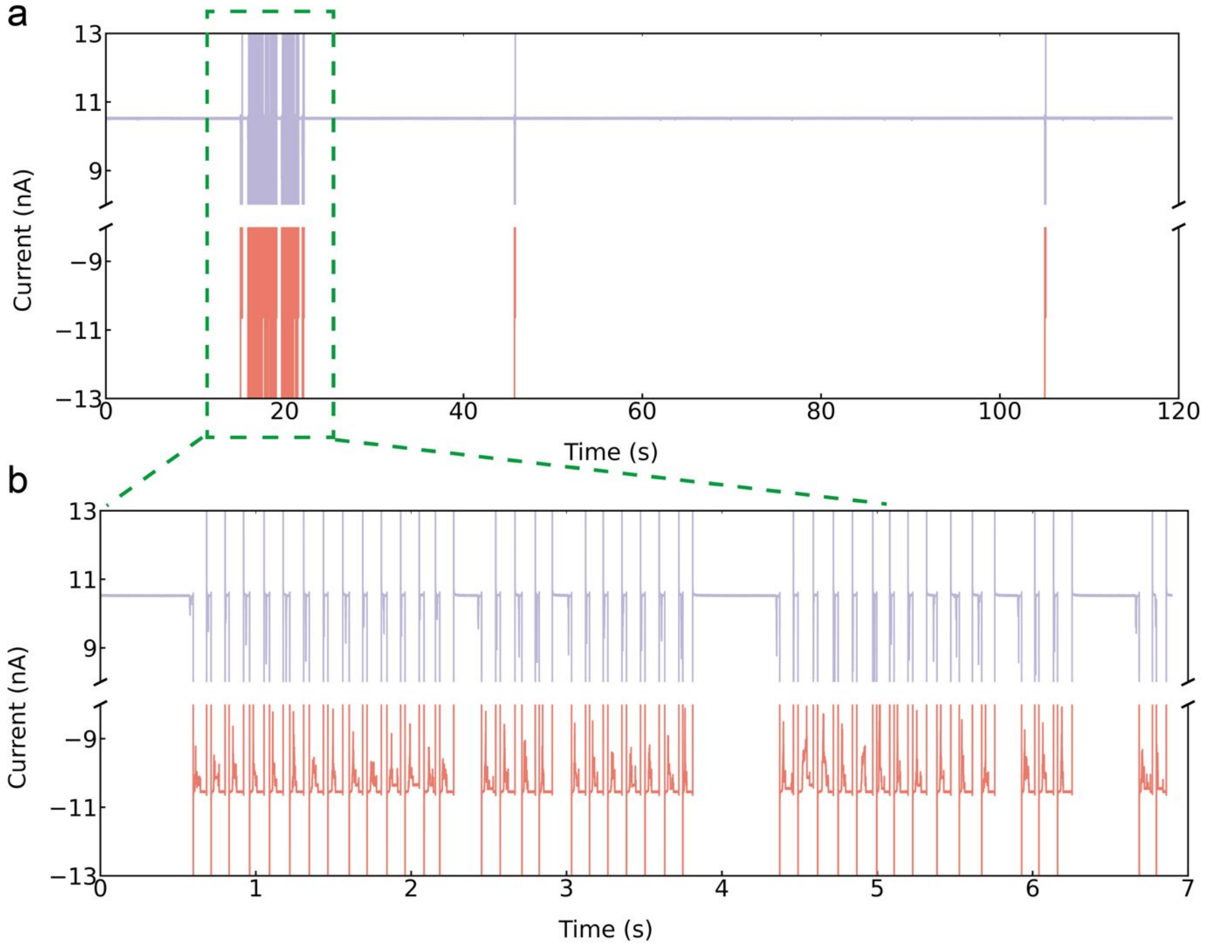

Figure S11. Example of the ping-pong current trace. a. Entire current trace of a 2 min ping-pong process; b. Enlarged view of the first ping-pong process (41-cycle recapture). Because of the low concentration we used, the translocation frequency of ping-pong experiments was quite low, ~ 3 events/min. This timescale was much longer than the time interval between two recapture events in ping-pong, which was usually less than 50 ms. Therefore, we could eliminate the possibility that a second DNA molecule was captured during the ping-pong process.

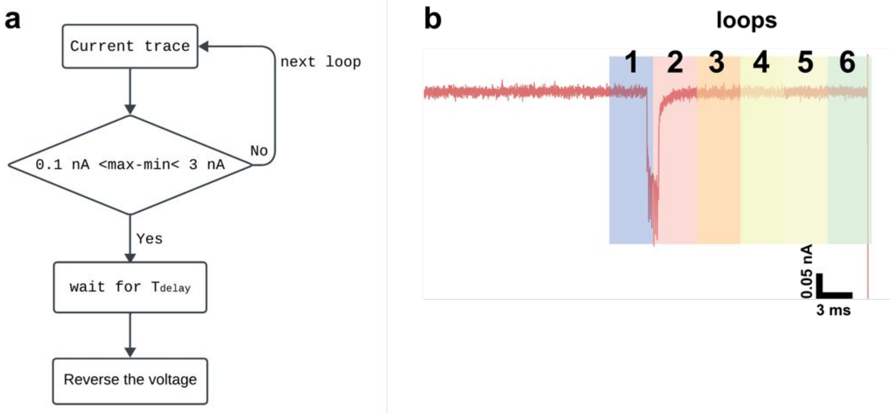

Figure S12. Illustration of ping-pong process. a. A flow chart describing the process of ping-pong; b. Corresponding current trace to explain the flow chart.

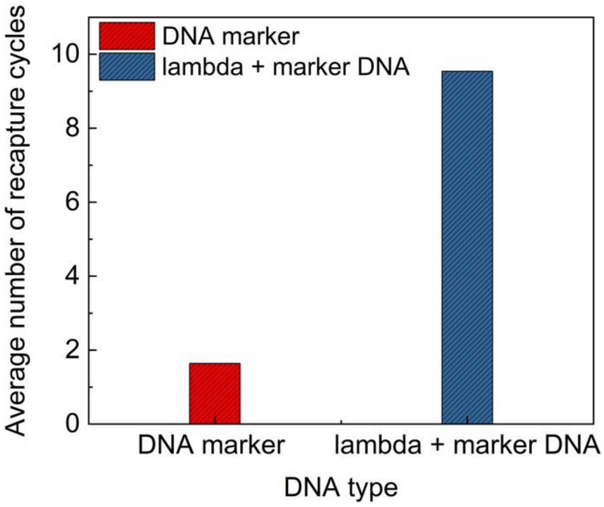

Figure S13. Average number of recapture cycles for DNA marker and lambda + marker DNA. The result was averaged from 50 ping-pong events respectively.

Reference:

1. Knowles, S. F. *et al.* Current Fluctuations in Nanopores Reveal the Polymer-Wall Adsorption Potential. *Phys. Rev. Lett.* **127**, 137801 (2021).
